# The MsmX ATPase plays a crucial role in pectin mobilization by *Bacillus subtilis*

**DOI:** 10.1371/journal.pone.0189483

**Published:** 2017-12-14

**Authors:** Mário J. Ferreira, Aristides L. Mendes, Isabel de Sá-Nogueira

**Affiliations:** UCIBIO, REQUIMTE, Departamento de Ciências da Vida, Faculdade de Ciências e Tecnologia, Universidade NOVA de Lisboa, Caparica, Portugal; Universidade do Minho, PORTUGAL

## Abstract

Carbohydrates from plant cell walls are often found as heteropolysaccharides intertwined with each other. For competitive advantage against other microorganisms, and ability to fully exploit available carbon and energy sources, *Bacillus subtilis* possesses a high number of proteins dedicated to the uptake of mono- and oligosaccharides. Here, we characterize transporter complexes, belonging to the ATP-binding cassette (ABC) superfamily, involved in the uptake of oligosaccharides commonly found in pectin. The uptake of these carbohydrates is shown to be MsmX-dependent, assigning a key role in pectin mobilization for MsmX, a multipurpose ATPase serving several distinct ABC-type I sugar importers. Mutagenesis analysis of the transmembrane domains of the AraNPQ MsmX-dependent importer revealed putative residues for MsmX interaction. Interestingly however, although MsmX is shown to be essential for energizing various ABC transporters we found that a second *B*. *subtilis* ATPase, YurJ, is able to complement its function when placed in *trans* at a different locus of the chromosome.

## Introduction

The plant cell wall is a highly complex structure, with a variable species- and tissue-dependent composition, and a major reservoir of carbohydrates in the form of cellulose, hemicellulose, and pectin [1, 2]. Cellulose is exclusively composed of D-glucose units, linked by β-1,4-glycosidic bonds and organized as linear parallel polymers connected via hydrogen bonds [1]. Hemicellulose and pectin are complex mixtures of branched polysaccharides composed of many different sugar monomers such as glucose, galactose, xylose, arabinose, mannose, rhamnose, and galacturonic acid [1, 3, 4].

Microorganisms have successfully developed concerted mechanisms for the degradation of these complex plant polysaccharides and the subsequent uptake of smaller sugar oligomers and monomers, which can be easily further metabolized. One such concerted mechanism is the *Bacillus subtilis* transport and utilization system for arabinan [5], a hemicellulose polysaccharide commonly found in low-lignin and pectin-rich substrates such as sugar beet pulp and citrus waste [4]. This bacterium in its natural environment–the soil or the gastrointestinal tract of animals [6, 7]–possesses two endo-α-1,5-arabinanases, AbnA and Abn2, responsible for breaking down the backbone of the homopolysaccharide arabinan [5, 8], releasing arabinose monomers and oligomers. Two intracellular α-L-arabinofuranosidases, AbfA and Abf2 [9], are accountable for further degrading the arabinooligosaccharides, after their uptake through two different types of transport systems. The AraE proton symporter is responsible for the uptake of arabinose and also plays a role in the transport of α-1,5-arabinobiose [10, 11]; the AraNPQ ABC-type system is responsible for the uptake of arabinooligosaccharides up to at least 4 arabinosyl units [11]. This system, along with the ABC-type maltodextrins importer MdxEFG, is energized by the multipurpose ATPase MsmX [11, 12]. However, MsmX together with another putative orphan ATPase, YurJ, are predicted to be the nucleotide-binding domain (NBD) that energizes five additional putative ABC-type importers of *B*. *subtilis* [13].

ABC transporters constitute one of the largest and most diverse transporter superfamilies and are found in all three domains of life (Archaea, Bacteria and Eukarya) [14, 15]. These transporters use the binding and hydrolysis of ATP to power the directional transport of a wide variety of substrates across membranes, ranging from ions to macromolecules, and all, importers or exporters, share a common structural organization: a modular architecture comprising two transmembrane domains (TMDs) that form the translocation pore and two NBDs that hydrolyze ATP [14–17]. Although multipurpose ABC ATPases, such as MsmX [11], have been described in other microorganisms, such as *Streptomyces lividans* [18], *Streptococcus pneumoniae* [19] and *Streptococcus suis* [20], the capability to interact with and energize multiple transporters is rather uncommon among this type of ATPases. In fact, to date the ability of a single ATPase to energize several ABC-type systems has been exclusively reported in bacterial sugar importers.

In previous work, we found that MsmX is crucial for the utilization of arabinan by *B*. *subtilis* and the results suggested the existence of other MsmX-dependent ABC importers involved in the uptake plant cell-wall oligosaccharides [11]. Here, we extend our investigation to the role of MsmX in the utilization of distinct plant cell wall polysaccharides by *B*. *subtilis* and the biological significance of sharing a single ATPase among multiple ABC transport systems. ABC-type I sugar importers responsible for the uptake of pectin-based polysaccharides were characterized and MsmX established as a key player in plant cell wall polysaccharides mobilization. Furthermore, using the AraNPQ importer as model, amino acid residues of the TMDs that are putatively important for the contacts with MsmX were identified by mutagenesis. In addition, we addressed the expression of both ATPases from *B*. *subtilis* MsmX and YurJ. We found that in the tested conditions YurJ is not present in the cell, thus we propose that expression of *yurJ* is subjected to post-transcriptional regulation.

## Materials and methods

### DNA manipulation and sequencing

Routine DNA manipulations were performed as described by Sambrook *et al*. [21]. All restriction enzymes were purchased from Thermo Fisher Scientific and used according to the manufacturers’ recommendations. PCR amplifications were carried out using Phusion High-Fidelity DNA Polymerase (Thermo Fisher Scientific). DNA from agarose gels and PCR products were purified with the illustra™ GFX™ PCR DNA and Gel Band Purification kit (GE Healthcare Life Sciences). All DNA ligations were performed using T4 DNA Ligase (Thermo Fisher Scientific). DNA phosphorylation was performed using T4 Polynucleotide Kinase (Thermo Fisher Scientific) and DNA dephosphorylation with FastAP Thermosensitive Alkaline Phosphatase (Thermo Fisher Scientific). Plasmids were purified using the NZYMiniprep kit (NZYTech). DNA sequencing was performed with the ABI PRISM BigDye^®^ Terminator Cycle Sequencing Kit (Applied Biosystems). The sequencing reaction was purified by gel filtration and resolved in an ABI 3730XL sequencer.

### Construction of *B*. *subtilis* strains

The generation of a *yurJ* clean deletion and in-frame clean deletion of *yesOPQ*, *ytcQ*, *cycB*, or *araQ*, in the *B*. *subtilis* chromosome, were obtained by overlapping PCR followed by allelic replacement using the pMAD vector according to the procedure described by Arnaud *et al*. [22]. Briefly, plasmids carrying the sequences of the regions immediately upstream and downstream of the gene(s) targeted for deletion were integrated into the chromosome of *B*. *subtilis* by a single recombination event, promoted by growth at a nonpermissive temperature (42 °C) for plasmid replication. A second single recombination event occurs during growth at a permissive temperature, thus allowing loss of the plasmid and substitution of the targeted allele without introduction of antibiotic resistance determinants. The ectopic regulated expression of *yurJ* or *msmX*, was carried out by placing each gene under the control of an IPTG-inducible promoter using the vector pDR111 (S1 Table) and subsequent integration at the *amyE* locus of the *B*. *subtilis* chromosome by a double recombination event. The creation of mutations in *araA* (E305A), *araP* (E208A, E205A and D213A), and *araQ* (D108A), leading to single amino acid substitutions, were obtained by overlapping PCR with mutagenic primers (S2 Table) followed by allelic replacement in the *B*. *subtilis* chromosome using the pMAD vector as described above. The insertion-deletion mutation of *galK* in the chromosome was performed by insertion of an erythromycin resistance cassette and concomitant partial deletion of *galK* by a double recombination event in the *B*. *subtilis* chromosome using a derivative of phagemid pBluescript II KS(+). The *msmX-his*_*6*_*-tag* and *yurJ-his*_*6*_*-tag* alleles were created by a single recombination event, at the respective locus of the *B*. *subtilis* chromosome, using plasmids harboring modified versions of *msmX* and *yurJ* that carry a His_6_-tag at the 3’ end. The Δ*msmX*::*spec* allele was created using pCm::Sp [23] to switch the chloramphenicol resistance marker of the Δ*msmX*::*cat* strain by a spectinomycin resistance marker. Transformations of *B*. *subtilis* strains were performed according to the method described by Anagnostopoulos and Spizizen [24]. All *B*. *subtilis* strains used in this study are listed in Table 1. A detailed description of plasmids (S1 Table), primers (S2 Table), and constructions (S1 Appendix), is found in the supporting information section.

**Table 1:** List of *B*. *subtilis* strains used or constructed during the course of this work.

Strain	Genotype	Source or Reference
**168T**^**+**^	Prototroph	F. E. Young
**IQB495** *	Δ*msmX*::*cat*	[11]
**IQB611** *	Δ*araNPQ*	[11]
**IQB618** *	Δ*yurJ*	pMJ14→168T^+^
**IQB622**	*msmX*-His_6_ *cat*	pGS1→168T^+^
**IQB623**	araP*E208A	pLB1→168T^+^
**IQB624**	araP*E205A	pMJ25→168T^+^
**IQB625**	araP*D213A	pMJ26→168T^+^
**IQB626**	araQ*D180A	pMJ27→168T^+^
**IQB627**	Δ*araQ* (4 codon 3’ deletion)	pMJ28→168T^+^
**IQB628** *	Δ*yesOPQ*	pMJ18→168T^+^
**IQB629** *	Δ*yesOPQ* Δ*araNPQ*	pMJ18→IQB611
**IQB630** *	Δ*galK*::*erm*	pMJ32→168T^+^ ^#^
**IQB631** *	Δ*galK*::*erm* Δ*araNPQ*	pMJ32→IQB611 ^#^
**IQB632** *	Δ*ytcQ*	pMJ33→168T^+^
**IQB633** *	Δ*ytcQ* Δ*yesOPQ*	pMJ33→IQB628
**IQB638** *	Δ*cycB*	pMJ38→168T^+^
**IQB639** *	Δ*cycB* Δ*araNPQ*	pMJ38→IQB611
**IQB641** *	ΔcycB araA*E305A	pLG31→IQB638
**IQB642** *	*amyE*::[Phyper-spank-*yurJ spec*] Δ*msmX*::*cat*	pMJ40→IQB495 ^#^
**IQB644**	*yurJ*-His_6_ *cat*	pMJ43→168T^+^
**IQB650**	Δ*msmX*::*spec*	pCm::Sp→IQB495
**IQB651**	Δ*msmX*::*spec yurJ*-His_6_ *cat*	pMJ43→IQB650
**IQB672**	*amyE*::[Phyper-spank *spec*] Δ*msmX*::*cat*	pDR111→IQB495 ^#^
**IQB673**	*amyE*::[Phyper-spank-*msmX spec*] Δ*msmX*::*cat*	pAM4→IQB495 ^#^

Arrows indicate transformation and point from donor DNA to the recipient strain.

# Transformation was carried out with linearized plasmid.

* The effect of these mutations in the utilization of several carbon sources is summarized in S3 Table.

### Growth conditions

*Escherichia coli* DH5α (Gibco-BRL) or *E*. *coli* XL1-Blue (Stratagene) were used for the construction of all plasmids. All *E*. *coli* strains were grown in liquid Lysogeny Broth (LB) medium [25] and on LB solidified with 1.6% (w/v) agar, where ampicillin (100 μg.mL^-1^), chloramphenicol (25 μg.mL^-1^), kanamycin (30 μg.mL^-1^) or tetracycline (12 μg.mL^-1^) were added as appropriate. *B*. *subtilis* strains were grown in liquid LB medium, LB solidified with 1.6% (w/v) agar or liquid SP medium [26], and chloramphenicol (5 μg.mL^-1^), kanamycin (10 μg.mL^-1^), erythromycin (1 μg.mL^-1^), spectinomycin (60 μg.mL^-1^), X-Gal (80 μg.mL^-1^) or IPTG (1 mM) were added as appropriate. Growth kinetics parameters of *B*. *subtilis* strains were determined in CSK minimal medium in the presence of 0.1% (w/v) of several carbon sources (D-(+)-glucose, L-(+)-arabinose, pectin (from apple) and D-(+)-galacturonic acid [Sigma-Aldrich Co]; α-1,5-arabinotriose, arabinan (sugar beet), galactan (ex. Lupin), polygalacturonic acid (PGA, from citrus pectin) and rhamnogalacturonan I (from potato pectic fiber) [Megazyme International Ireland]), as described by Ferreira and Sá-Nogueira [11]. The optical density (OD_600nm_) of growing cultures was used for the determination of the doubling time for each strain in the presence of the tested sugars as sole carbon and energy sources.

### RNA extraction

*B*. *subtilis* 168T^+^ was grown in CSK minimal medium supplemented with 0.1% (w/v) of glucose, arabinose or arabinotriose, as previously described for growth kinetics parameters and doubling time determinations [11]. When the OD_600nm_ of growing cultures reached 0.7–0.8, cells were collected by centrifuging 1.4 mL of each culture at 6000*g* and 4 °C for 5 minutes. Total RNA extraction was performed using the Absolutely Total RNA Miniprep kit (Agilent Technologies) according to the manufacturer’s instructions and using RNase-free technique. The integrity of RNA was analyzed in a 1% (w/v) agarose gel in TBE 1x. DNA contamination of RNA samples was assessed by PCR using primers ARA422 and ARA423 (S2 Table). Total RNA was quantified using a Nanodrop™ 1000 Spectrophotometer (Thermo Fisher Scientific) and stored in 10 μL aliquots at -80 °C.

### RT-qPCR experiments and data analyses

The primers used for RT-qPCR experiments–ARA583 and ARA584 (16S gene), ARA638 and ARA639 (*yurJ*), and ARA640 and ARA641 (*msmX*)–were designed with the help of the online tool Primer3Plus (http://primer3plus.com/cgi-bin/dev/primer3plus.cgi). Primer efficiency was assessed using the Rotor-Gene SYBR^®^ Green PCR Kit (QIAGEN) and RT-qPCR experiments were performed using the SensiFAST™ SYBR No-ROX One-Step Kit (Bioline), both in a Rotor-Gene 6000 (Corbett) real-time cycler. RT-qPCR experiments were performed according to the kit manufacturer’s instructions, using 40 ng of total RNA and 0.1 μM of each primer, in a volume of 12.5 μL. Statistical analyses were performed with GraphPad Prism (version 5.00) using *C*_*t*_ values obtained from three independent assays. *p* values were determined using an unpaired *t* test.

### Protein extracts of *B*. *subtilis* and Western Blot analysis

*B*. *subtilis* strains IQB622, IQB644, and IQB651 were grown in CSK minimal medium supplemented with 0.1% (w/v) of glucose, arabinose or arabinotriose, as previously described for growth kinetics parameters and doubling time determination [11]. Cells of growing cultures were collected as described for RNA extraction (see above). The collected cells were resuspended in 100 μL of Lysis Buffer (500 mM KCl, 20 mM HEPES K^+^, pH 7.6, 10 mM EDTA, 1 mM DTT, 10% glycerol). Lysozyme (1 mg.mL^-1^) was added and the mixture was incubated for 10 min at 37 °C, followed by three cycles of freezing in liquid nitrogen and thawing for 5 min at 37 °C. 10 mM PMSF and 7.5 U of Benzonase® Nuclease (Sigma-Aldrich Co.) were added followed by an incubation for 15 min at 37 °C. Total protein content for each extract was determined using Bio-Rad Protein Assay (Bio-Rad Laboratories, Inc.). 20 μg of total protein from each extract and 1 μg of purified MsmX-His_6_ were loaded in a 12.5% SDS-PAGE and run at constant electrical current (30 mA) for 50 min. The fractionated proteins were then electrotransfered into a nitrocellulose membrane (0.45 μm; Bio-Rad), for 90 min at constant voltage (100 V) and 4 °C. Protein-blocking to the membrane was then performed using 20 mL of a powdered milk solution in TBS-Tween (5% w/v) for 30 min at room temperature with mild shaking. The membrane was then washed with TBS-Tween for 15 min at room temperature with mild shaking, followed by overnight incubation, at 4 °C, with 10 mL of primary antibody solution (mouse monoclonal Anti-6X His-tag® antibody [HIS.H8; Abcam], diluted 1:1000 in PBS-Tween with 4% w/v powdered milk). All subsequent incubation and wash steps were performed at room temperature with mild shaking. The membrane was washed twice with 10 mL of TBS-Tween, for 10 min, followed by incubation with 10 mL of secondary antibody solution (HRP-conjugated goat anti-mouse IgG antibody [Jackson ImmunoResearch Europe Ltd.], diluted 1:10000 in TBS-Tween with 4% w/v powdered milk) for 30 min. Finally, the membrane was washed three times with 10 mL of TBS-Tween solution. All subsequent steps were performed in a dark room. Blots were developed using 1 mL Western Lightning™ ECL Pro kit (PerkinElmer). Amersham Hyperfilm plates (GE Healthcare) were exposed to luminescence for 30 sec inside a closed Hypercassette Autoradiography Cassette (GE Healthcare). The membrane was then stripped and reprobed with a second primary antibody solution (mouse monoclonal Anti-σ^70^ [from *E*. *coli*; a gift from Fujita M.], diluted 1:1000 in PBS-Tween with 4% w/v powdered milk) for loading control. The same secondary antibody was used as described above. Blots were developed using 1 mL Western Lightning™ ECL Pro kit (PerkinElmer). Amersham Hyperfilm plates (GE Healthcare) were exposed to luminescence for 3 min inside a closed Hypercassette Autoradiography Cassette (GE Healthcare).

## Results

### Identification of carbohydrates imported via MsmX-dependent systems

In this work, we used an *msmX*-null mutant strain to determine the impact of this mutation on the ability of *B*. *subtilis* cells to grow on different sugars, commonly found in the hemicellulosic fraction of plant cell wall, as sole carbon and energy source. Both the wild-type strain (168T^+^) and the *msmX*-null mutant strain (IQB495, S1 Fig) were grown in the presence of pectin, galactan, rhamnogalacturonan type I, polygalacturonan and galacturonic acid, and the results are summarized in Table 2.

**Table 2:** Growth of a *B*. *subtilis msmX*-null mutant in the presence of different mono- and polysaccharides. Doubling time (min) of different strains in liquid minimal medium (CSK) using pectin, galactan, rhamnogalacturonan I, polygalacturonan or galacturonic acid as sole carbon and energy source. Results are the averages of three independent assays and their respective standard deviations. NG, no growth.

	168T^+^ (wild-type)	IQB495 (Δ*msmX*::*cat*)
**Pectin 0.1%**	153.1 ± 12.4	NG
**Galactan 0.1%**	130.7 ± 6.5	NG
**Rhamnogalacturonan I 0.1%**	189.3 ± 15.3	603.9 ± 32.6
**Polygalacturonan 0.1%**	123.5 ± 4.4	365.6 ± 44.7
**Galacturonic Acid 0.1%**	77.3 ± 0.6	79.5 ± 1.1

The ability to utilize pectin was completely abolished in the *msmX*-null mutant when compared to the wild-type. Because this polysaccharide is highly complex, branched, and composed by various sugars, mainly arabinose, galactose, rhamnose and galacturonic acid, this result strongly suggests that MsmX is involved in the uptake of most of these sugars. Growth in the presence of galactan is also abolished when a functional MsmX is missing (Table 2), assigning this ATPase as a critical player in the utilization of galactan. Since galactose is imported by the arabinose proton-symporter AraE [10, 27], MsmX is likely to be responsible for energizing an ABC-type importer dedicated to the uptake of galactooligosaccharides resulting from the extracellular degradation of galactan.

The absence of a functional MsmX also had a significant negative impact on the growth of *B*. *subtilis* in minimal medium supplemented with rhamnogalacturonan type I or polygalacturonan (Table 2). Unlike the complete loss of growth observed when arabinan [11] or galactan were used as sole carbon and energy source, a slower but steady growth rate was detected for the *msmX-*null strain in the presence of either rhamnogalacturonan type I or polygalacturonan. Interestingly, the increase of the doubling-time in the mutant strain (IQB495) when compared to the wild-type was very similar for both polysaccharides, about 3-fold (Table 2). Since galacturonic acid is the major component of both polysaccharides, the effect of the Δ*msmX*::*cat* mutation in the uptake of this monosaccharide was also assessed and the results show that the utilization of galacturonic acid is MsmX-independent (Table 2). Thus, the negative impact in the growth kinetics parameters observed in the presence of rhamnogalacturonan type I or polygalacturonan is most probably due to the existence of at least one MsmX-dependent ABC-type importer responsible for the uptake of galacturonic acid oligomers and/or rhamnose-galacturonic acid disaccharides but not galacturonic acid.

### Functional characterization of ABC-type sugar importers

**Fig 1:**
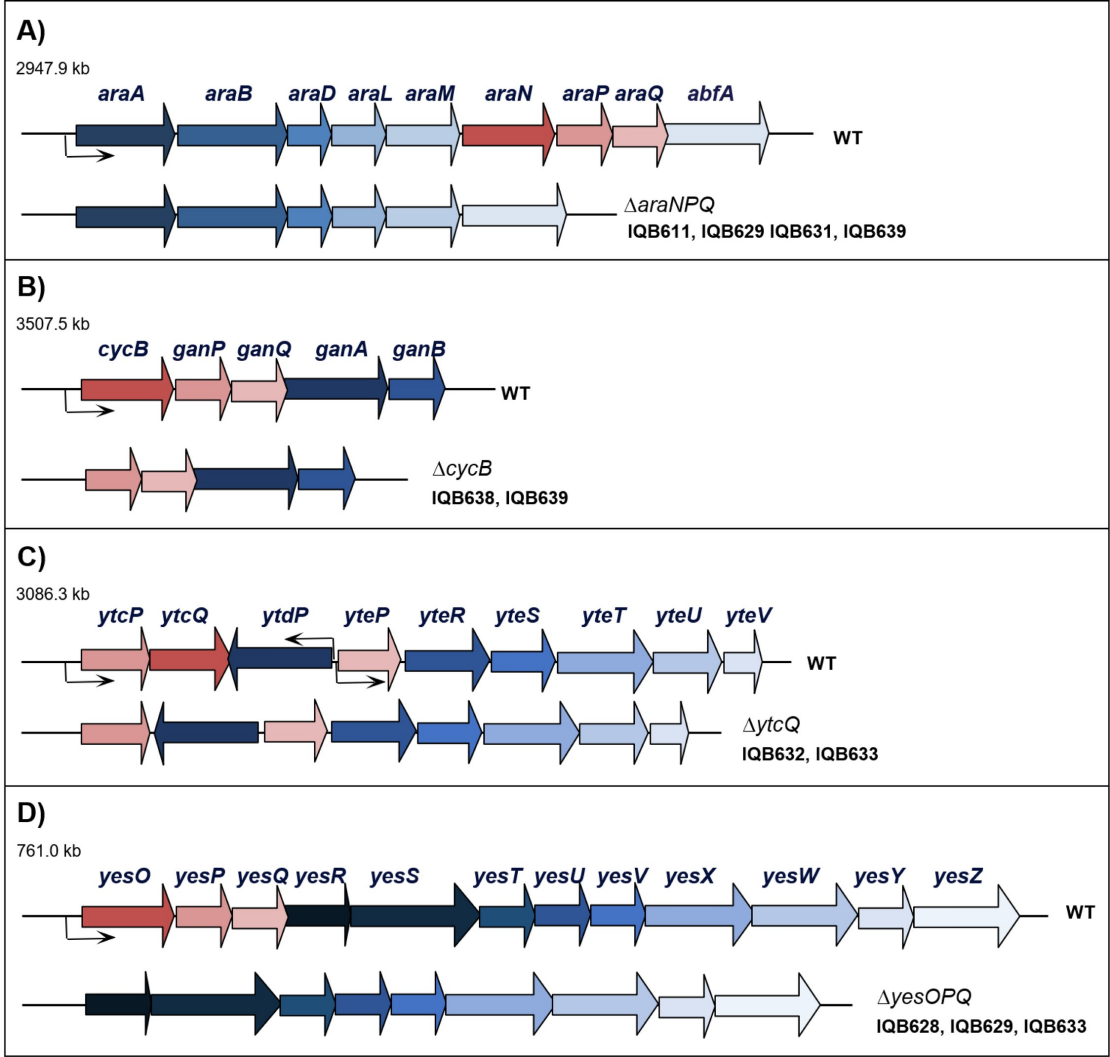
Organization of the *araABDLMNPQ-abfA*, *cycB-ganPQAB*, *ytcPQ-ytdP-ytePRSTUV* and *yesOPQRSTUVWXYZ* loci of the wild-type (WT) and mutant *B*. *subtilis* strains. The location of the four regions is indicated in kilobase pairs. The genes are represented by arrows pointing in the direction of transcription. The constructs bearing the mutations used in this work are displayed below each region, and the strains harboring each mutation are indicated in front of the construct. The in-frame deletions generated by allelic replacement, Δ*araNPQ*, Δ*cycB*, Δ*ytcQ*, and Δ*yesOPQ*, are depicted below the *araABDLMNPQ-abfA* (A), *cycB-ganPQAB* (B), *ytcPQ-ytdP-ytePRSTUV* (C), and *yesOPQRSTUVWXYZ* (D) loci, respectively. All constructions are described in Materials and Methods and supporting information.

In a previous work by our group [11], it was suggested that AraNPQ was only responsible for the uptake of linear α-1,5-linked arabinooligosaccharides, and nonlinear α-1,2- and α-1,3-linked oligomers were transported by an unidentified MsmX-dependent system, because an *araNPQ* in-frame deletion (Fig 1A) partially abolished growth on branched arabinan while prevented growth on debranched arabinan. The *cycB-ganPQAB* operon (Fig 1B), encodes a putative β-galactosidase (GanA), a putative arabinogalactan endo-β-1,4-galactanase (GanB), an ABC-type solute-binding protein (SBP), CycB, two transmembrane domains (TMD), GanP and GanQ, and was proposed to be involved in the utilization of galactan [28, 29]. Since the CycB-GanPQ system (formerly YvfKLM) was also suggested to be energized by MsmX [13], and the arabinose proton symporter AraE is able to transport arabinose, xylose and galactose [10, 27], we targeted this transporter as the system potentially responsible for the uptake of nonlinear arabinooligosaccharides.

An in-frame deletion mutation of the *cycB* gene was generated by allelic replacement (Fig 1B), and the physiological effect of this mutation in the utilization of arabinan by *B*. *subtilis* strains IQB638 (Δ*cycB*) and IQB639 (Δ*cycB* Δ*araNPQ*) was assessed by the determination of growth kinetics parameters (Table 3). Similarly to that observed for the Δ*araNPQ* mutant (188.8 ± 15.3 min; IQB611 [11]), only a marginal increase in the doubling time of the Δ*cycB* mutant was detected (IQB638; Table 3), when compared do the wild-type strain grown on arabinan (149.0 ± 6.8 min; strain 168T^+^ [11]). Nonetheless, the ability of the double Δ*cycB* Δ*araNPQ* mutant (IQB639) to grow on the same substrate was completely abolished (Table 3), suggesting that the putative MsmX-dependent transporter for nonlinear arabinooligosaccharides was indeed CycB-GanPQ.

**Table 3:** Uptake of arabinan in *B*. *subtilis*. Doubling time (minutes) for different strains in liquid minimal medium (CSK) using glucose or arabinan as sole carbon and energy source. Results are the averages of three independent assays and their respective standard deviations. NG, no growth.

	IQB638 (Δ*cycB*)	IQB639 (Δ*cycB*Δ*araNPQ*)	IQB630 (Δ*galK*::*erm*)	IQB631 (Δ*araNPQ* Δ*galK*::*erm*)
**Glucose 0.1%**	52.6 ± 0.1	52.1 ± 1.2	45.8 ± 1.2	53.9 ± 1.7
**Arabinan (branched) 0.1%**	168.7 ± 39.4	NG	300.4 ± 28.1	NG

However, we later found out that the branched sugar beet arabinan (Megazyme International), contained a level of galactose 5-fold higher than that disclosed (less than 4% (w/w) of galactose). Thus, to investigate if the growth observed in the Δ*araNPQ* mutant was owed to the presence of galactose, or most likely galactooligosaccharides, and not due to nonlinear arabinooligosaccharides, an insertion-deletion mutation of the galactose kinase gene (*galK*) was generated (S1 Fig). In the resulting Gal^**-**^ strains, IQB630 (Δ*galK*::*erm*) and IQB631 (Δ*galK*::*erm* Δ*araNPQ*), an increase in the doubling time was observed for the single mutant and the double mutant is impaired to grow in the presence of branched arabinan as the sole carbon and energy source (Table 3). These results confirmed that the branched arabinan utilized is highly contaminated with galactose/galactooligosaccharides and consequently responsible for maintaining growth of *B*. *subtilis* when a functional AraNPQ system is unavailable. Additionally, these results implicate that the CycB-GanPQ system plays a role in the uptake of galactooligosaccharides, as previously suggested [28, 29].

CycB was previously described as a cyclodextrin-binding protein [30]. However, during the course of this work a recent study showed that CycB (renamed GanS) was able to bind sugar oligomers with three and four galactosyl units [31]. Thus, we tested the impact of deleting the *cycB* gene in the ability to utilize galactan. The obtained results show that the Δ*cycB* mutant (IQB638) displays a 2-fold increase in the doubling time when galactan is the sole carbon and energy source, when compared to the wild-type strain (Table 4). Therefore, CycB is involved in the utilization of galactan by *B*. *subtilis*, and is likely to be part of a putative CycB-GanPQ ABC-type importer responsible for the uptake of galactooligosaccharides released by the enzymatic breakdown of galactan by the extracellular endo-β-1,4-galactanase GanB.

**Table 4:** Uptake of galactan in *B*. *subtilis*. Doubling time (minutes) for different strains in liquid minimal medium (CSK) using glucose or galactan as sole carbon and energy source. Results are the averages of three independent assays and their respective standard deviations. NG, no growth.

	168T^+^ (wild-type)	IQB638 (Δ*cycB*)	IQB639 (Δ*cycB* Δ*araNPQ*)	IQB641 (ΔcycB araA*E305A)
**Glucose 0.1%**	55.5 ± 1.3	52.6 ± 0.1	52.1 ± 1.2	54.2 ± 2.1
**Galactan 0.1%**	130.7 ± 6.5	258.3 ± 29.6	NG	532.8 ± 2.2

In addition, we also tested a double Δ*cycB* Δ*araNPQ* mutant strain (IQB639) in the presence of galactan and its ability to grow was completely lost. To confirm that the observed growth of the Δ*cycB* mutant in the presence of galactan was due to the presence of arabinose, we constructed an Ara^-^ strain by substituting the key catalytic residue E305 of L-arabinose isomerase (AraA) by an alanine. This mutation was previously shown to completely block the isomerization of L-arabinose to L-ribulose, thus preventing its metabolism [32]. When grown in the presence of galactan, the ΔcycB araA*E305A strain (IQB641) presented an extremely high doubling time (Table 4), which confirms that the growth observed in the Δ*cycB* mutant strain (IQB638) is most probably due to the presence of arabinose and/or arabinosyl oligomers.

Polygalacturonan and rhamnogalacturonan type I are the two major components of pectin. The former is a linear chain of D-galacturonic acid units, while the latter has a backbone composed of alternating L-rhamnose and D-galacturonic acid units highly decorated with mainly arabinans and galactans as side chains [1, 33]. Ochiai *et al*. [33] previously showed that the genes encoding the putative YesOPQ and YtcQP-YteP ABC-type importers, comprising the predicted solute-binding proteins YesO and YtcQ, and two transmembrane domains each, YesPQ and YtcP-YteP, respectively, were induced in the presence of rhamnogalacturonan type I. Since these transporters were proposed to be energized by MsmX [13], and this ATPase was shown to be important for the utilization of these polysaccharides by *B*. *subtilis* (Table 2), we constructed in-frame Δ*ytcQ* and Δ*yesOPQ* mutations (Fig 1C and 1D, respectively) and assessed their effect in the uptake of polygalacturonan and rhamnogalacturonan type I. The results presented in Table 5 showed that both mutations have a negative impact in the utilization of the two polysaccharides, moreover this effect was larger in the Δ*ytcQ* mutant (IQB632) than in the Δ*yesOPQ* strain (IQB628). These results indicate that YtcQP-YteP is involved in the uptake of polygalacturonan and rhamnogalacturonan, and suggest that YesOPQ may also play a role in the utilization of these polysaccharides. However, the double mutant strain (Δ*ytcQ* Δ*yesOPQ*, IQB633) displayed no increase in the doubling time in the presence of either substrate when compared to the single Δ*ytcQ* mutant (Table 5). This observation may indicate that Δ*yesOPQ* has only a marginal effect in the uptake of these substrates, which is likely to be negligible in the double Δ*ytcQ* Δ*yesOPQ* mutant since the Δ*ytcQ* mutation reveals a significantly higher impact in the utilization of both polysaccharides.

**Table 5:** Uptake of pectin-based polysaccharides in *B*. *subtilis*. Doubling time (minutes) for different strains in liquid minimal medium (CSK) using glucose, pectin, polygalacturonan or rhamnogalacturonan I as sole carbon and energy source. Results are the averages of three independent assays and their respective standard deviations.

	168T^+^ (wild-type)	IQB628 (Δ*yesOPQ*)	IQB632 (Δ*ytcQ*)	IQB633 (Δ*ytcQ* Δ*yesOPQ*)
**Glucose 0.1%**	55.5 ± 1.3	54.5 ± 0.4	53.3 ± 1.8	54.7 ± 3.4
**Pectin 0.1%**	153.1 ± 12.4	147.7 ± 14.7	139.0 ± 19.9	154.7 ± 27.8
**Polygalacturonan 0.1%**	123.5 ± 4.4	153.0 ± 19.0	186.8 ± 16.8	180.6 ± 17.8
**Rhamnogalacturonan I 0.1%**	189.3 ± 15.3	225.5 ± 23.6	495.9 ± 54.3	491.9 ± 61.8

Additionally, for both polysaccharides, the doubling time of the double Δ*ytcQ* Δ*yesOPQ* mutant (Table 5) was lower than that observed for the Δ*msmX*::*cat* mutant (Table 2). This observation was expected for rhamnogalacturonan I, since the uptake of the oligosaccharides released from the breakdown of its arabinan and galactan side chains is MsmX-dependent, but polygalacturonan is a linear homopolysaccharide composed of galacturonic acid units. Nevertheless, our results, together with the previous observations by Ochiai *et al*. [33], indicate that the multitask ATPase MsmX most probably has a role in energizing the YesOPQ and YtcQP-YteP systems (Table 2 and Table 5).

### Functional analysis of *yurJ*

The *B*. *subtilis* chromosome encodes two highly similar ATPases, MsmX and YurJ (>55% amino acid identity), belonging to sub-family 5a of ABC-type importers associated to carbohydrate uptake [13]. MsmX also displays a high amino acid identity (>40%) to the MalK ATPase [34] belonging to the *E*. *coli* maltose/maltodextrins ABC-type importer. The complete MalEFGK_2_ transporter structure determination revealed the amino acids responsible for the contacts between the TMDs and the NBDs [35]. An alignment of the primary sequence of the three proteins, MsmX, YurJ and MalK, showed that most of the MalK amino acids contacting the TMDs are conserved in both MsmX and YurJ (S2 Fig). Furthermore, the similarity between both *B*. *subtilis* ATPases suggests the possibility of YurJ being able to associate to MsmX-energized ABC transporters.

We have previously showed that depletion of MsmX in a *msmX*-null mutant (strain IQB495) impairs the utilization of arabinose oligomers, which is AraNPQ-dependent, thus indirectly demonstrating that YurJ is unable to functionally substitute MsmX in the conditions tested [11]. Nevertheless, we constructed a deletion of *yurJ* in the *B*. *subtilis* chromosome (Δ*yurJ*; strain IQB618, Table 1 and S1 Fig) and tested its ability to grow on arabinotriose as the sole carbon and energy source. The results shown in Table 6 indicated that this deletion has no negative impact in the ability to grow on arabinotriose when compared to the wild-type strain 168T^+^ (98.2 ± 10.0 min [11]).

**Table 6:** Effect of *yurJ* deletion and complementation analysis of *msmX* deletion in *B*. *subtilis*. Doubling time (min) of different strains in liquid minimal medium (CSK) using glucose or arabinotriose as sole carbon and energy source. Results are the averages of three independent assays and their respective standard deviations. ^#^ IPTG was added to a final concentration of 1 mM. NG, no growth.

	IQB618 (Δ*yurJ*)	IQB672 (Δ*msmX*::*cat amyE*::[Phyper-spank *spec*])	IQB673 (Δ*msmX*::*cat amyE*::[Phyper-spank-*msmX spec*])	IQB642 (Δ*msmX*::*cat amyE*::[Phyper-spank-*yurJ spec*])
**Glucose 0.1%**	50.4 ± 5.5	59.8 ± 0.7 ^#^	59.1 ± 2.0 ^#^	52.9 ± 3.2 ^#^
**Arabinotriose 0.1%**	92.8 ± 10.9	NG ^#^	107.6 ± 3.9 ^#^	94.6 ± 0.5 ^#^

To confirm that MsmX is the sole ATPase responsible for energizing the importer AraNPQ, we placed the *msmX* allele under the control of an IPTG inducible promoter (P_hyper-spank_) at the *amyE* locus of the chromosome in a Δ*msmX* genetic background, strain IQB673 (Table 1 and S1 Fig). By comparison to the negative control strain, IQB672, the ectopic controlled expression of *msmX* was able to re-establish the capacity to utilize arabinotriose in a *msmX*-null genetic background (Table 6). Interestingly, although *yurJ* is not able to energize AraNPQ in a *msmX*-null background (strain IQB495; Table 1), when the *yurJ* allele is placed at the *amyE* locus of the chromosome (strain IQB642, Table 6 and S1 Fig) complementation of the *msmX* deletion is attained. These observations indicate that YurJ is in fact capable to functionally complement MsmX and energize the uptake of arabinooligosaccharides by AraNPQ, moreover suggest that in the conditions tested YurJ is not present in the cell when the *yurJ* allele is located in its natural chromosomal genetic context.

### A post-transcriptional regulatory mechanism controls *yurJ* expression

**Fig 2:**
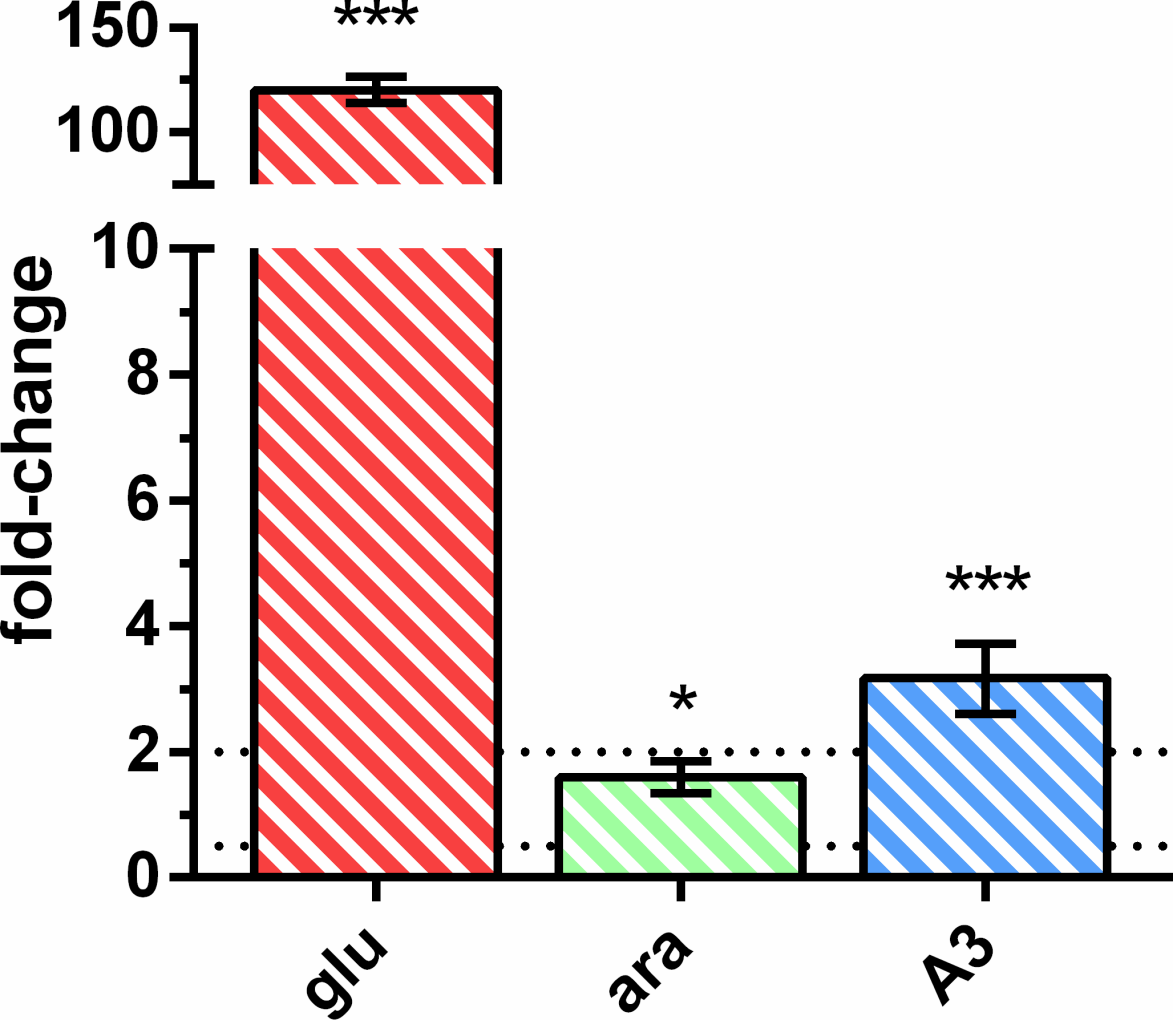
mRNA level of *yurJ* versus *msmX* in the *B*. *subtilis* wild-type strain. The results represent the relative expression of *yurJ* versus *msmX*. Upper and lower relative expression threshold of 2.0 and 0.5, respectively, are represented by dotted lines. (glu, *red*-striped bar), (ara, *green*-striped bar) and (A3, *blue*-striped bar) represent the presence of 0.1% of glucose, arabinose or arabinotriose, respectively. Primers ARA638/ARA639 and ARA640/ARA641 were used for *yurJ* and *msmX*, respectively. Fold-change was normalized using primers ARA583 and ARA584 for the 16S gene. Statistical analyses were performed with GraphPad Prism (version 5.00) using *C*_*t*_ values obtained from three independent assays. *p* values were determined using an unpaired *t* test (^ns^, non-significant difference; *, *p* < 0.05; **, *p* < 0.01; ***, *p* < 0.001).

Since YurJ is able to energize the importer AraNPQ in an *msmX*-null mutant strain when expressed in *trans* under the control of an IPTG-inducible promoter, but not from its own locus, we analyzed its expression at the transcriptional level in the wild-type strain. The expression level of both *yurJ* and *msmX* was determined in the same conditions described above by measuring the mRNA levels of both transcripts using quantitative reverse transcription polymerase chain reaction (RT-qPCR). The obtained data were used to calculate gene expression fold-change and the results are summarized in Table 7 and Fig 2.

**Table 7:** Quantification of *yurJ* and *msmX* mRNA level in the *B*. *subtilis* wild-type strain. The results represent the fold-change of the expression in the target conditions (_T_) versus the control conditions (_C_). (glu), (ara) and (A3) indicate the presence of 0.1% of glucose, arabinose or arabinotriose, respectively. Primers ARA638 and ARA639 were used for *yurJ* and primers ARA640 and ARA641 for *msmX*. Fold-change was normalized using primers ARA583 and ARA584 for the 16S gene. Statistical analyses were performed with GraphPad Prism (version 5.00) using *C*_*t*_ values obtained from three independent assays. *p* values were determined using an unpaired *t* test (^ns^, non-significant difference; *, *p* < 0.05; **, *p* < 0.01; ***, *p* < 0.001).

	*yurJ*		*msmX*	
	(glu)_T_	(A3)_T_	(glu)_T_	(A3)_T_
**(ara)**_**C**_	0.993 ± 0.054^ns^	1.782 ± 0.323*	0.013 ± 0.001***	0.899 ± 0.051^ns^
**(glu)**_**C**_	-	1.794 ± 0.311**	-	68.120 ± 3.683***

**Fig 3:**
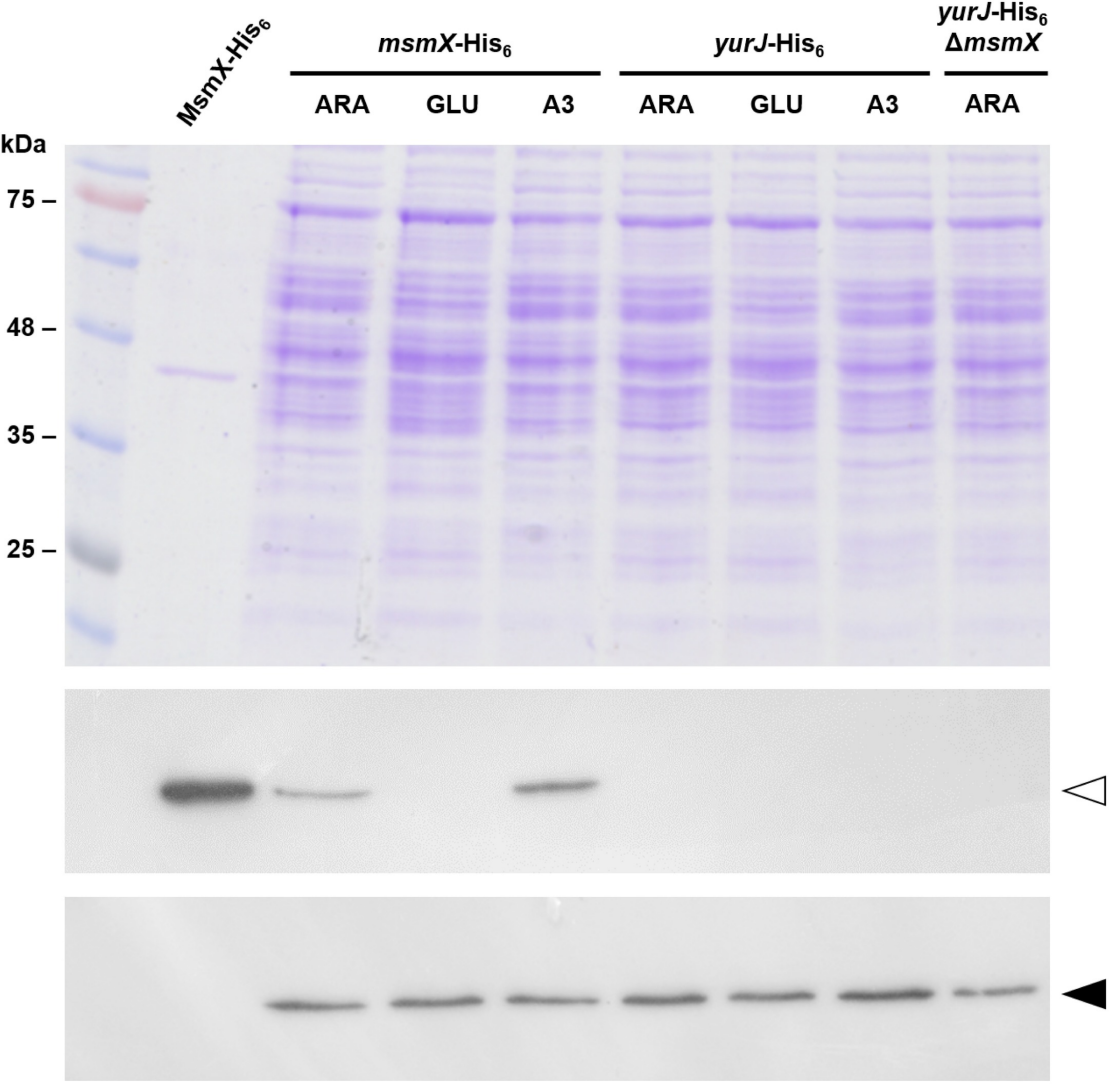
MsmX and YurJ detection by Western Blot. *Top panel*, SDS-PAGE (12.5%) with a molecular weight marker (NZYColour Protein Marker II) and samples used for Western Blot (20 μg of total extract and 1 μg of purified MsmX-His_6_ were loaded); *Middle panel*, Autoradiography plate after His-tag detection using an anti-His_6_-specific antibody; *Bottom panel*, Autoradiography plate after σ^A^ detection using an anti-σ^70^-specific antibody. *msmX*-His_6_, *yurJ*-His_6_, and *yurJ*-His_6_ Δ*msmX* represent strains IQB622, IQB644, and IQB651 respectively. MsmX-His_6_ (42.4 kDa) and σ^A^ (42.9 kDa) are indicated by open or closed arrowheads, respectively. Extracts obtained from cells grown in minimal medium supplemented with arabinose (ARA), glucose (GLU) or arabinotriose (A3) as described in Materials and Methods.

The *msmX* and *yurJ* mRNA level was similar in the presence of both arabinose and arabinotriose, however in the presence of glucose *msmX* expression is repressed about 75-fold while the level of *yurJ* mRNA was not subjected to glucose repression. These observations correlate with previous results that place *msmX*, but not *yurJ*, in the CcpA regulon [36–38]. Furthermore, when directly comparing the transcript levels from both genes it was clear that there was no significant difference in their expression in the presence of arabinose or arabinotriose (Fig 2). In fact, when arabinotriose is used in growth assays a slightly higher expression level (about 3-fold) was detected for *yurJ* when compared to *msmX* (Fig 2). These results clearly show that in these conditions *yurJ* is expressed at the transcriptional level in its natural genetic context in the *B*. *subtilis* chromosome suggesting the existence of a post-transcriptional regulatory mechanism responsible for the inability of YurJ to substitute MsmX in a *msmX*-null mutant. Thus, in order to determine if YurJ is translated, modified versions of both ATPases were constructed by fusing a C-terminal His_6_-tag to *msmX* (IQB622) and *yurJ* (IQB644) in their own loci, as result of a single recombination event. In addition, a modified version of *yurJ-his*_*6*_ was also generated in a *msmX*-null background (IQB651). The accumulation of MsmX-His_6_ and YurJ-His_6_ in cells grown in the same conditions used in RT-qPCR experiments was detected by Western blot analysis. Similar amounts of total protein extracts from the three strains, grown in the presence of arabinose, and/or glucose or arabinotriose, were used for the detection of MsmX-His_6_ (42.4 kDa) and YurJ-His_6_ (42.2 kDa), respectively. Additionally, a protein extract of the wild-type strain (168T^+^) grown in CSK with arabinose was used as negative control and purified recombinant MsmX-His_6_ used as positive control for His-tag detection (Fig 3).

The data confirm the presence of MsmX in the cells when grown in minimal medium with either arabinose or arabinotriose, however in identical conditions YurJ was absent (Fig 3). As result of the strong glucose-mediated repression of *msmX*, in accordance to the results obtained by RT-qPCR, no MsmX is detected when glucose is present in the medium (Fig 3). Thus, by combining the RT-qPCR data and Western Blot analysis, we show that although *msmX* and *yurJ* are being similarly expressed in the presence of arabinotriose, the latter translation product is not detected. Furthermore, in a *msmX*-null background, or in conditions in which MsmX is not present, YurJ remains undetectable. Together the results indicate that in minimal medium with either glucose, arabinose or arabinotriose as sole carbon and energy source, *yurJ* is transcribed but not translated.

### Identification of key residues for the interaction between MsmX and the TMDs of AraNPQ

**Fig 4:**
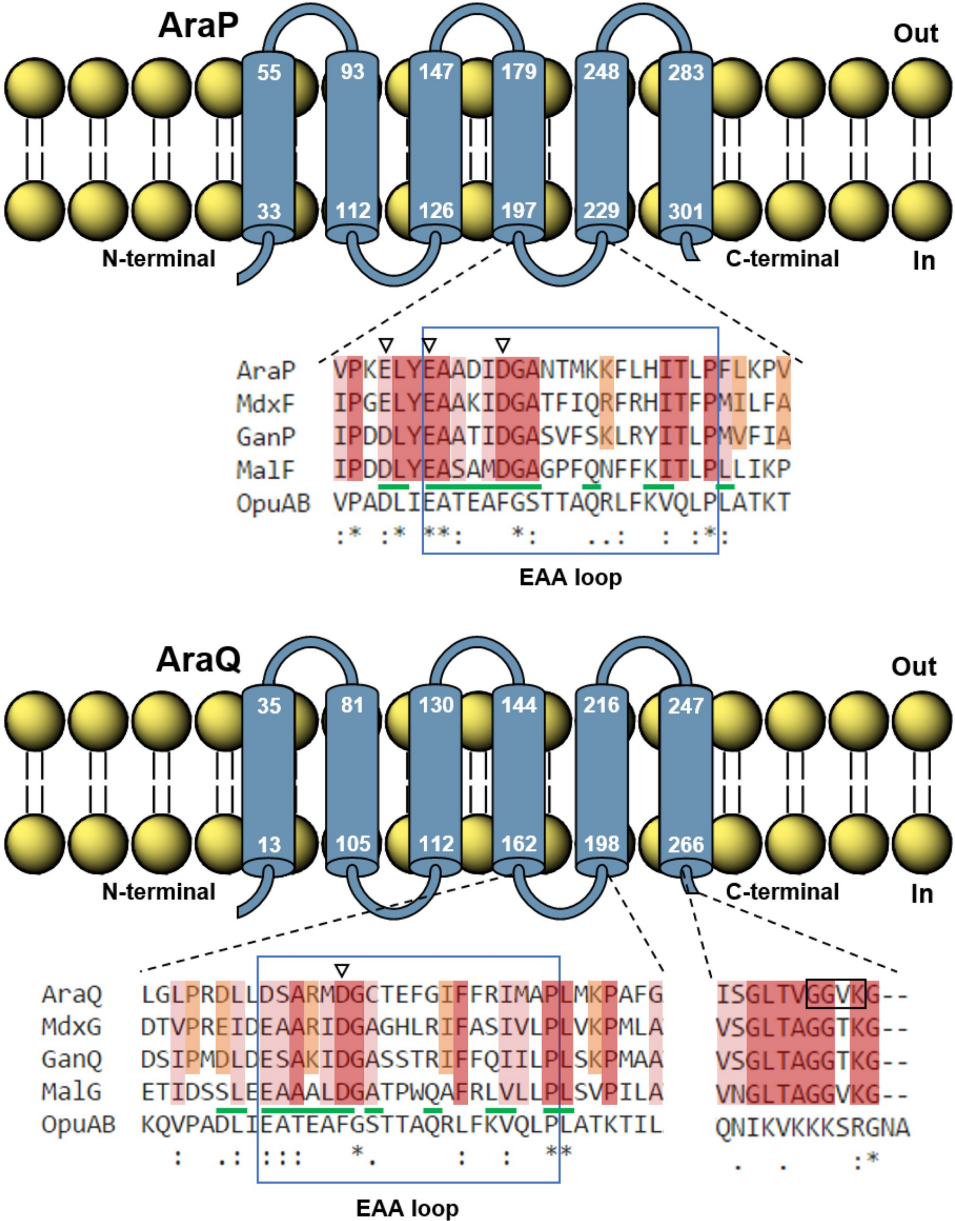
The TMDs from the AraNPQ-MsmX system. Membrane-spanning domains in AraP (top) and AraQ (bottom) were predicted using TMpred (http://www.ch.embnet.org/software/TMPRED_form.html). The position of the first and last amino acid of each membrane-spanning domain is indicated. Alignments of the TMDs from the *B*. *subtilis* Ara, Mdx, Gan, and OpuA and the *E*. *coli* Mal ABC transporters were obtained using Clustal Omega (http://www.ebi.ac.uk/Tools/msa/clustalo/) and are partially shown. Identical (´*´) and similar (´.´ or ´:´) amino acids are indicated. Gaps in the amino acid sequences inserted for alignment optimization are indicated by a dash (–). The “EAA” sequence motifs in the last cytoplasmic loop are boxed. Identical residues between the four TMDs belonging to sugar transporters are highlighted in *red*. Similar residues between the four TMDs belonging to sugar transporters are highlighted in *pink*. Identical or similar residues between the three TMDs belonging to sugar transporters from *B*. *subtilis* are highlighted in *orange*. Residues selected for mutagenesis in AraP and AraQ are indicated by open arrowheads. MalF and MalG residues known to establish contacts with the MalK dimer are underlined in *green*. Residues selected for deletion in the C-terminal end of AraQ are boxed. Accession numbers: AraP (P94529), AraQ (P94530), MdxF (O06990), MdxG (O06991), GanP (O32261), GanQ (O07011), OpuAB (P46921), MalF (P02916), and MalG (P68183).

Most ABC-type systems of *B*. *subtilis* possess its own dedicated NBD, but a few, particularly those involved in sugar uptake, share a single ATPase among them [11–13]. Systems relying on the same ATPase must have similar amino acid (and/or structural) determinants that mediate NBD-TMD interactions. In ABC importers, these interactions occur mainly, but not exclusively, between specific conserved motifs: the “Q loop” of the NBD, which is characterized by a highly-conserved glutamine residue; and the “EAA” sequence motif (consensus EAAX_3_GX_9_IXLP) in the last cytoplasmic loop of the TMD [14, 39, 40]. Based on the complete structure the *E*. *coli* ABC-type maltose importer (MalEFGK_2_) and identification of the amino acids directly responsible for the NBD-TMD contacts [35]. Sequence alignments between the TMDs (Fig 4) and NBDs (S3 Fig) of MalEFGK_2_, *B*. *subtilis* systems energized by MsmX (AraNPQ, MdxEFG, and CycB-GanPQ) and the glycine betaine importer OpuA of *B*. *subtilis* [41], allowed the identification of residues in AraP and AraQ which are potentially involved in the contacts with MsmX. Similarities between the MsmX-dependent systems and MalK from *E*. *coli* and differences between these and the OpuA system possessing its own NBD were key features for determining which amino acids should be targeted. Single alanine substitutions of residues E205, E208 and D213 in AraP and D180 in AraQ were generated and their effect in the functionality of AraNPQ was inferred by the impact in arabinotriose uptake (Table 8).

**Table 8:** Effect of mutations in AraP and AraQ in the uptake of α-1,5-arabinotriose. Doubling time (min) of different strains in liquid minimal medium (CSK) using glucose or arabinotriose as sole carbon and energy source. Results are the averages of three independent assays and their respective standard deviations.

	IQB623 (araP*E208A)	IQB624 (araP*E205A)	IQB625 (araP*D213A)	IQB626 (araQ*D180A)	IQB627 (Δ*araQ*)
**Glucose 0.1%**	56.5 ± 1.2	53.1 ± 0.1	53.2 ± 1.0	54.9 ±1.7	56.1 ± 2.3
**Arabinotriose 0.1%**	114.0 ± 4.8	94.1 ± 5.0	357.0 ± 25.3	149.3 ± 12.8	472.8 ± 22.6

These results showed that the single E205A mutation in AraP had no effect on the growth rate of *B*. *subtilis*, while the E208A substitution resulted in a small negative impact in the uptake of arabinotriose, an increase of about 10% in the doubling time of strain IQB623 (araP*E208A; Table 8) when compared to the wild-type (98.2 ± 10.0 min [11]). This is in accordance with the results published by Mourez *et al*. [42] for the *E*. *coli* maltose transporter, where for each of the TMDs, a single alanine substitution of the residue homologous to E208 from AraP (E401 in MalF and E190 in MalG) had little to no impact in the uptake of maltose. Although E205 and E208 from AraP are still likely to establish contacts with MsmX, they may not be essential for TMD-NBD interaction. On the other hand, the D213A mutation in AraP severely impaired the uptake of arabinotriose as shown by the increase in the doubling time approximately 3.6-fold. Likewise, although not as adversely, the homologous D180A substitution in AraQ had also a negative impact in the growth rate of *B*. *subtilis* in the presence of arabinotriose (Table 8).

In the *E*. *coli* maltose transporter the Q loop of MalK establishes additional contacts with the C-terminal end of MalG. This tail, which is not found in MalF, inserts itself along the MalK dimer interface and its four terminal amino acids (-GVKG) interact with residues from the Q loop of both NBDs [35]. Interestingly, the primary sequence of this C-terminal tail from MalG is highly conserved in AraQ, MdxG and GanQ but not in the MsmX-independent OpuAB (Fig 4). Since this feature was only found in systems that are associated with MsmX we deleted the four terminal amino acids of AraQ and assessed the impact of their absence in the uptake of arabinotriose. This deletion resulted in a severe increase of almost 5-fold in the doubling time of *B*. *subtilis* strain IQB627 (Δ*araQ*) when compared to that of the wild-type (Table 8). In *B*. *subtilis* this C-terminal tail feature is not found in other sub-families of ABC-type transporters (with the exception of sub-family 5a) and might be a requirement for the interaction between the ATPase MsmX and the TMDs of systems it energizes. However, we cannot exclude the possibility that the small deletion generated in AraQ caused a destabilization of the protein leading to its degradation.

## Discussion

**Fig 5:**
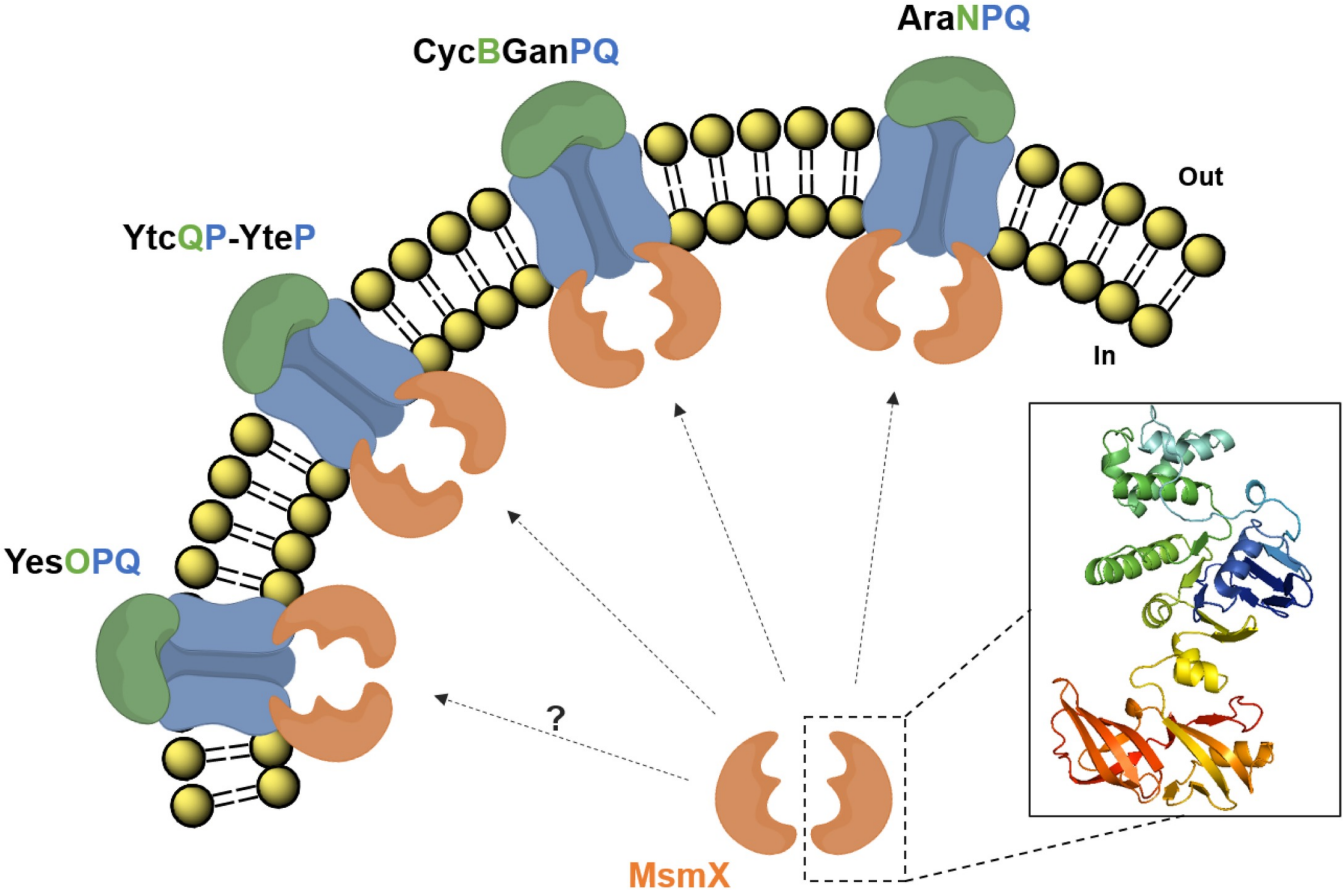
*B*. *subtilis* ABC-type sugar importers energized by MsmX and involved in pectin mobilization. AraNPQ, CycB-GanPQ, YtcQP-YteP, and most likely YesOPQ, are energized by MsmX and play a role in the uptake of carbohydrates resulting from the breakdown of pectin. AraNPQ and CycB-GanPQ are respectively responsible for the uptake of arabino- and galactooligosaccharides from the side chains of pectin; YtcQP-YteP and YesOPQ are involved in the uptake of galacturonic acid oligomers and/or rhamnose-galacturonic acid disaccharides. A representation of the predicted structure for an MsmX (Accession number: P94360) monomer, obtained using I-TASSER (http://zhanglab.ccmb.med.umich.edu/I-TASSER/; version 3.0), is shown.

ABC transporter genes are the most frequent class of protein coding genes found in the *B*. *subtilis* genome [43]. Three ABC-type I sugar importers have been identified in *B*. *subtilis* that are energized by the same ATPase, MsmX: AraNPQ, responsible for the uptake of arabinooligosaccharides [11]; MdxEFG, involved in the transport of maltodextrins [12]; and CycB-GanPQ, which is the major transporter of galactooligosaccharides [31]. During the course of this work Watzlawick *et al*. [31] indirectly demonstrated that MsmX energizes CycB-GanPQ, since its presence was shown to be essential for induction of the *cycB-ganPQAB* operon in the presence of galactan. The results presented here also suggest that the previously uncharacterized YtcQP-YteP and YesOPQ systems are energized by MsmX, as proposed by the work of Quentin *et al*. [13]. Furthermore, we provide evidence that these systems play a role in the transport of polygalacturonan (or galacturonic acid oligomers) and rhamnogalacturonan type I (or rhamnose-galacturonic acid disaccharides), as previously hypothesized by Ochiai *et al*. [33]. According to a microarray analysis on the utilization of carbohydrates by an alkaliphilic *Bacillus* sp., genes with high identity to *yesOPQ* and *ytcQP-yteP* were shown to have increased expression levels in the presence of pectin [44], and further bioinformatics analysis [45], support the role of these transporters in the uptake of polygalacturonan and rhamnogalacturonan. Our work shows that the utilization of arabinan, galactan, rhamnogalacturonan type I and polygalacturonan by *B*. *subtilis* is completely or partly MsmX-dependent, thus establishing this ATPase as a key player in the uptake of pectin-rich substrates. MsmX is now associated to four different ABC-type I sugar importers involved in the mobilization of pectin (Fig 5).

To apprehend the reason why carbohydrate ABC-type I importers share a common ATPase, in contrast to the majority of ABC-type importers and exporters encoded in the *B*. *subtilis* chromosome, we may speculate in this particular case it may reflect the fact that in plant biomass sugars are often found as heteropolysaccharides, i.e., sugars like arabinose, galactose or xylose are usually present in the same substrates. Unlike glucose, which is the premier source of energy for most heterotrophic organisms and effectively downregulates the expression of most genes associated with the utilization of other sugars, a clear hierarchy among those is not as well defined. Although a preference of one carbohydrate over another has been reported for both *B*. *subtilis* [37, 46] and *E*. *coli* [47] that might not be the case for sugars transported via the MsmX-dependent ABC importers. Being often simultaneously available, oligomers of arabinose, galactose and galacturonic acid, and rhamnose-galacturonic acid disaccharides are likely to be transported at the same time. Thus, a single ATPase capable of serving the various carbohydrate-specific transport systems, like MsmX, may be a more effective strategy for the cell to respond quickly, adapt and proliferate in a specific ecologic niche.

Additionally, we found that in an engineered *B*. *subtilis* strain an additional ATPase (YurJ) encoded in its genome is able to functionally replace MsmX when ectopically expressed under the control of an inducible promoter. Moreover, the results presented here show that YurJ is not present in the cell in the conditions tested (Fig 3), despite the presence of similar levels of *yurJ* and *msmX* mRNA (Fig 2). Thus, a post-transcriptional regulatory mechanism, is likely to control the synthesis of YurJ in the conditions tested. It has been reported in proteomic studies of the *B*. *subtilis* cytosol that YurJ is present in the cell in certain conditions [48]. Furthermore, a condition-dependent transcriptome analysis of *B*. *subtilis* [49] indicated that *yurJ* is co-transcribed together with a putative antisense RNA (S1254) located downstream from the *frlBONMD* genes (formerly *yurPONML*) involved in the utilization of Amadori products (fructosamines) [50]. A longer transcription unit including *yurJ* was also detected (*frlB*-*frlO*-*frlN*-*frlM*-*frlD*-S1254-*yurJ*), and the putative antisense RNA overlaps and pairs to the convergent regulatory gene of the operon, *frlR* [49]. All these observations suggest the involvement of the antisense RNA (S1254) in preventing *yurJ* translation by an unknown mechanism.

In the genome of *B*. *subtilis*, no genes encoding putative ABC-type importers are found close to the gene encoding MsmX, but *yurJ* is located in the close proximity of the *frlONM* genes, which encode a putative ABC-type importer. Although no association between YurJ and *flrONM* has been established [50], due to its genomic context this ATPase is likely to energize its neighboring system. Nevertheless, we may hypothesize that interchangeability between MsmX and YurJ may occur in specific conditions which allow translation of YurJ. In fact, two ABC ATPases of *Streptococcus mutans*, MsmK and MalK, are capable of substituting each other and energize both transporters for raffinose/stachyose and for maltodextrins, even though they are encoded by genes located in the vicinity of the remaining components of their partner ABC transporter [51].

In order to understand why some ATPases are able to bind to various ABC-type systems when most are restricted to interactions with a single transporter, several of the residues expected to mediate the TMD-NBD contacts between AraPQ and MsmX were mutated and their effect on sugar uptake assessed. An E208A substitution in AraP displayed a small negative impact in the uptake of arabinotriose, in accordance with previously published results that report a similar substitution in MalF of the *E*. *coli* maltose transporter also showing a very limited impact [42]. Likewise, a homologous mutation in MalG had an identical effect, and only when both glutamate residues in MalF and MalG were substituted a significant decrease in the uptake of maltose was observed. Accordingly, a *B*. *subtilis* strain harboring an E208A and a D175A substitution in AraP and AraQ, respectively, is expected to be severely impaired in the transport of arabinooligosaccharides. In contrast to the E208A mutation in AraP, single D213A and D180A substitutions in AraP and AraQ, respectively, displayed a significant negative impact in the uptake of arabinotriose. Based on the crystal structure obtained for the complete maltose transporter of *E*. *coli* [35] and the sequence homology with the AraNPQ system of *B*. *subtilis*, we hypothesize that both D213 in AraP and D180 in AraQ are likely to establish more contacts with MsmX than those expected for E208 of AraP, and consequently more important for maintaining the correct assembly of all components in the AraNPQ-MsmX system. Another putative structural feature in the ABC systems energized by MsmX is a highly-conserved C-terminal tail in the second TMD of each system. This feature is also found in MalG of the *E*. *coli* maltose system in which the tail is inserted along the ATPase dimer interface, possibly contributing to keep the dimer in a correct position [35]. In fact, upon deletion of this C-terminal tail in AraQ, a severe decrease in the growth rate of *B*. *subtilis* in the presence of arabinotriose was observed (Table 8). This tail is not found in ABC systems belonging to other sub-families, which further emphasizes its putative significance in the specific binding between MsmX and the ABC sugar importers of *B*. *subtilis*. In order to gain insight into AraPQ-MsmX interactions attempts to solve the crystal structure of MsmX are currently in progress.

## Supporting information

S1 Table List of plasmids used or constructed during this work. (DOCX)

S2 Table List of all oligonucleotides used during this study. (DOCX)

S3 Table Growth of B. subtilis strains in the presence of different carbon sources. The effect of the depicted mutations in the ability to grow in liquid minimal medium (CSK) supplemented with a single carbon/energy source is represented by ‘++’ (normal growth rate), ‘+’ (slightly to moderately decreased growth rate), and ‘-’ (strongly decreased growth rate or no growth). ‘NA’, no data available. * Grown in the presence of 1mM IPTG. Wild-type is *B*. *subtilis* 168T^+^, and all other strains have a 168T^+^ background.
(DOCX)

S1 Fig Organization of the *msmX, amyE*, and *galK* loci of the wild-type (WT) and mutant *B. subtilis* strains. The location of the three regions is indicated in kilobase pairs. The genes are represented by arrows pointing in the direction of transcription. The constructs bearing the mutations used in this work are displayed below each region, and the strains harboring each mutation are indicated in front of the construct. The mutations generated by the ectopic insertion of *msmX* or *yurJ* under control of an inducible promoter are represented at the *amyE* locus. The insertion-deletion mutation created by a deletion in *msmX* followed by the insertion of a chloramphenicol resistance cassette (*cat*) is shown below the *msmX* locus. The insertion-deletion mutation created by a deletion in *galK* followed by the insertion of an erythromycin resistance cassette (*erm*) is shown below the *galK* locus. All constructions are described in Materials and Methods and supporting information.
(TIF)

S2 Fig MsmX, YurJ and MalK protein sequence alignment. The alignment between *B*. *subtilis* MsmX and YurJ, and *E*. *coli* MalK was obtained using Clustal Omega (http://www.ebi.ac.uk/Tools/msa/clustalo/). Identical (´*´) and similar (´.´ or ´:´) amino acids are indicated. Gaps in the amino acid sequences inserted for alignment optimization are indicated by a dash (–). Conserved ABC ATPase motifs (Walker A, Q loop, Signature motif, Walker B, D loop, and H loop) are boxed. Identical residues between the three ATPases are highlighted in *red*. Identical residues between MsmX and YurJ are highlighted in *pink*. MalK residues involved in interactions with the TMDs of the *E*. *coli* maltose transporter are underlined in *green*. Accession numbers: MsmX (P94360), YurJ (O32151), and MalK (P68187).
(TIF)

S3 Fig Sequence alignment of three ABC-type ATPases. The alignment between *B*. *subtilis* MsmX and OpuAA, and *E*. *coli* MalK was obtained using Clustal Omega (http://www.ebi.ac.uk/Tools/msa/clustalo/). Identical (´*´) and similar (´.´ or ´:´) amino acids are indicated. Gaps in the amino acid sequences inserted for alignment optimization are indicated by a dash (–). MalK residues involved in interactions with the TMDs of the *E*. *coli* maltose transporter are highlighted in *red* (identical in MsmX), *pink* (similar in MsmX), or *orange* (not conserved in MsmX). Accession numbers: MsmX (P94360), MalK (P68187), and OpuAA (P46920).
(TIF)

S1 Appendix Construction of plasmids and *B. subtilis* strains. (DOCX)
