# Supplementary material for: The MsmX ATPase plays a crucial role in pectin mobilization by Bacillus subtilis
Source: PLoS One. 2017 Dec 14;12(12):e0189483. doi: 10.1371/journal.pone.0189483 (PMC5730181; doi:10.1371/journal.pone.0189483)
Supplement: S1 Table — (DOCX) [file pone.0189483.s001.docx]

**Table S1 – List of plasmids used or constructed during this work.**

| **Plasmid** | **Relevant features** | **Source or Reference** |
| --- | --- | --- |
| **pBluescript II KS(+)** | Integrative phagemid with a *lacZ* gene for blue/white selection of clones, *bla* | Stratagene |
| **pET30a(+)** | Expression vector with an inducible T7 promoter for the expression of proteins in *E. coli*, *kan* | Novagen |
| **pDG1731** | Plasmid used as a source of erythromycin resistance cassette, *bla*, *erm*, *spec* | [52] |
| **pDR111** | Integrative plasmid which allows the insertion of genes under the control of Phyper-spank in the *amyE* locus, *bla*, *spec* | David Rudner |
| **pLitmus29** | Integrative plasmid with a *lacZ* gene for blue/white selection of clones, *bla* | New England Biolabs |
| **pMAD** | Plasmid used for allelic replacement in Gram-positive bacteria, *bla*, *erm* | [22] |
| **pCm::Sp** | Plasmid used for replacement of a chloramphenicol resistance marker by a spectinomycin resistance marker, *spec* | [23] |
| **pUC18** | Integrative plasmid with a *lacZ* gene for blue/white selection of clones, *bla* | [53] |
| **pMJ14** | Integrative plasmid used for the construction of *yurJ* in-frame deletion mutants, *bla*, *erm* | This work |
| **pMJ18** | Integrative plasmid used for the construction of *yesOPQ* in-frame deletion mutants, *bla*, *erm* | This work |
| **pMJ21** | pET30a(+)-based vector with a truncated version of *msmX*-His_6_, *kan* | This work |
| **pMJ22** | pET30a(+)-based vector for the expression of *msmX*-His_6_, *kan* | This work |
| **pMJ23** | pBluescript II KS(+)-based plasmid used as a source of erythromycin resistance cassette, *bla*, *erm* | This work |
| **pMJ25** | Integrative plasmid used for the construction of *araP* E205A mutants, *bla*, *erm* | This work |
| **pMJ26** | Integrative plasmid used for the construction of *araP* D213A mutants, *bla*, *erm* | This work |
| **pMJ27** | Integrative plasmid used for the construction of *araQ* D180A mutants, *bla*, *erm* | This work |
| **pMJ28** | Integrative plasmid used for the construction of *araQ* mutants with an in-frame deletion of 4 C-terminal codons, *bla*, *erm* | This work |
| **pMJ31** | pBluescript II KS(+)-based plasmid harboring the coding region of *galK*, bla | This work |
| **pMJ32** | Integrative plasmid used for the construction of *galK* null-mutations, *bla*, *erm* | This work |
| **pMJ33** | Integrative plasmid used for the construction of *ytcQ* in-frame deletion mutants, *bla*, *erm* | This work |
| **pMJ38** | Integrative plasmid used for the construction of *cycB* in-frame deletion mutants, *bla*, *erm* | This work |
| **pMJ40** | Integrative plasmid used for the insertion of *yurJ* under the control of Phyper-spank in the *amyE* locus, *bla*, *spec* | This work |
| **pMJ43** | Integrative plasmid used for the fusion of a C-terminal Histidine tag to *yurJ*, *bla*, *cat* | This work |
| **pAM4** | Integrative plasmid used for the insertion of *msmX* under the control of Phyper-spank in the *amyE* locus, *bla*, *spec* | This work |
| **pGS1** | Integrative plasmid used for the fusion of a C-terminal Histidine tag to *msmX*, *bla*, *cat* | This work |
| **pJL1** | pLitmus29-based plasmid used as a source of erythromycin resistance cassette, *bla*, *erm* | This work |
| **pLB1** | Integrative plasmid used for the construction of *araP* E208A mutants, *bla*, *erm* | This work |
| **pLG31** | Integrative plasmid used for the construction of *araA* E305A mutants, *bla*, *erm* | Godinho, unpublished results |
| **pMS38** | pLitmus29-based plasmid used as a source of chloramphenicol resistance cassette, *bla*, *cat* | [54] |

[52] Guérout-Fleury AM, Frandsen N, Stragier P (1996) Plasmids for ectopic integration in *Bacillus subtilis*. Gene 180(1-2): 57-61.

[53] Yanisch-Perron C, Vieira J, Messing J (1985) Improved M13 phage cloning vectors and host strains: nucleotide sequences of the M13mp18 and pUC19 vectors. Gene 33(1): 103-119.

[54] Zilhão R, Serrano M, Isticato R, Ricca E, Moran CP, Henriques AO (2004) Interactions among CotB, CotG, and CotH during assembly of the *Bacillus subtilis* spore coat. J Bacteriol 186(4): 1110-1119.
