# Supplementary material for: The MsmX ATPase plays a crucial role in pectin mobilization by Bacillus subtilis
Source: PLoS One. 2017 Dec 14;12(12):e0189483. doi: 10.1371/journal.pone.0189483 (PMC5730181; doi:10.1371/journal.pone.0189483)
Supplement: S2 Table — (DOCX) [file pone.0189483.s002.docx]

**Table S2 – List of all oligonucleotides used during this study.**

| **Oligonucleotide** | **Sequence (5’→3’)** | **Complementary sequence** |
| --- | --- | --- |
| **ARA55** | CTATCATTAGTACGTATCTTTTGTATT | *araA* |
| **ARA422** | TATTGAGCTCTCAGGGATAGATATCAAATCG | *msmX* |
| **ARA423** | CATCGGTACCATTCTGAGATTTTCAATAGC | *msmX* |
| **ARA517** | TCCCCGATATCACAATCCAGG | *yurJ* |
| **ARA518** | AGCCTCTTCCGTTTCCGCATCGAATGTTAATGAAGCCATCAGAAAAGTCCC | *yurJ* |
| **ARA519** | GGGACTTTTCTGATGGCTTCATTAACATTCGATGCGGAAACGGAAGAGGCT | *yurJ* |
| **ARA520** | ATGACCGATATCCGTCTTCCC | *yurJ* |
| **ARA544** | GGATGTCATATGGCTGAATTGCGG | *msmX* |
| **ARA545** | CCGGTTTTTTTGCTCGAGTCGGATTCTCAC | *msmX* |
| **ARA566** | GGGTTTGCCGGTGCAGGAGATTGGAAGAC | *araA* |
| **ARA567** | GTCTTCCAATCTCCTGCACCGGCAAACCC | *araA* |
| **ARA573** | CATACGCATAAATCTGCATG | *araA* |
| **ARA583** | TCGCGGTTTCGCTGCCCTTT | *16S* |
| **ARA584** | AAGTCCCGCAACGCGCGCAA | *16S* |
| **ARA630** | TACATGAAGGATCCTGCCAAGGC | *araP* |
| **ARA631** | CAGCGGCTGCGTACAGCTCT | *araP* |
| **ARA632** | AGAGCTGTACGCAGCCGCTG | *araP* |
| **ARA633** | TTGGAAGGCCATGGGCATATTGC | *araP* |
| **ARA634** | ATGTGCTGACCATGGGCATCATCG | *araQ* |
| **ARA637** | CGGGATCCATGCCTCTTGTG | *araQ* |
| **ARA638** | TTCGATCCGGCTTCATATTC | *yurJ* |
| **ARA639** | CGTTCGCTTGAAACACTGAA | *yurJ* |
| **ARA640** | CCGAAGCCTGAAATCAAAAA | *msmX* |
| **ARA641** | ACAAAGGCTCATCCATCAGG | *msmX* |
| **ARA642** | AAACACGCGTTGGACCATATCC | *yesO* |
| **ARA645** | CCGGATCCTCCAAAATTGTATGCC | *yesQ* |
| **ARA652** | CTGTGCCATCTGCAACACTCCTTTTTAGTAACAAATCTTCTTCAAACCAATCCC | *yesO* |
| **ARA653** | GGGATTGGTTTGAAGAAGATTTGTTACTAAAAAGGAGTGTTGCAGATGGCACAG | *yesQ* |
| **ARA667** | GCTTCGTACAGCGCTTTCGGC | *araP* |
| **ARA668** | GCCGAAAGCGCTGTACGAAGC | *araP* |
| **ARA669** | GTATTCGCGCCGGCTATATCAGC | *araP* |
| **ARA670** | GCTGATATAGCCGGCGCGAATAC | *araP* |
| **ARA671** | CGTACAGCCGGCCATCCTTGC | *araQ* |
| **ARA672** | GCAAGGATGGCCGGCTGTACG | *araQ* |
| **ARA673** | CGTTTATAGGATCCCGCTGACC | *araQ* |
| **ARA674** | CATCACACGTTTCCTCCTTCATTACCCTACCGTCAGGCCGGAGATAAA | *araQ* |
| **ARA675** | TTTATCTCCGGCCTGACGGTAGGGTAATGAAGGAGGAAACGTGTGATG | *araQ* |
| **ARA676** | GCCAACGCGTTTGGCAGATTGT | *araQ* |
| **ARA714** | GTGTTCATCGATTCAACTCC | *cycB* |
| **ARA718** | ATGAGAGCTCTGCAGCATATGATCTGC | *galK* |
| **ARA719** | TTTGGTACCAGGCCATACTCAAGCAGC | *galK* |
| **ARA733** | TTCGCTAGATGCTTATCGC | *ytcQ* |
| **ARA734** | TGGGTTTTTCCAATGTTATTTTGCTTGCACCCTCCATTTGTTTCCCATCGTACC | *ytcQ* |
| **ARA735** | GGTACGATGGGAAACAAATGGAGGGTGCAAGCAAAATAACATTGGAAAAACCCA | *ytcQ* |
| **ARA736** | TCAGACGCGTTGAAATATCG | *ytcQ* |
| **ARA741** | CTTGTGTCGACAGGGAATTGCTG | *msmX* |
| **ARA742** | CCCTTGCATGCGGTTTGATTCTGAG | *msmX* |
| **ARA779** | GCTTTCTCTTTATTTTCCGCTGTGTGCCATTTTCATTGATTGTCACCC | *cycB* |
| **ARA780** | GGGTGACAATCAATGAAAATGGCACACAGCGGAAAATAAAGAGAAAGC | *cycB* |
| **ARA781** | TCTAACGCGTAGAAAGCCAAG | *cycB* |
| **ARA820** | ACCAAGTCGACTCCAGTGACG | *yurJ* |
| **ARA821** | GCAATGCATGCATCGACCACC | *yurJ* |
| **ARA831** | CTCAGAAAGATGGCGAAACAGG | *yurJ* |
| **ARA832** | CTAGTGGTGGTGGTGGTGGTGATACACAGCCTCTTCCGTTTCCG | *yurJ* |

Restriction sites in the primer sequences are underlined.
