# Supplementary material for: The MsmX ATPase plays a crucial role in pectin mobilization by Bacillus subtilis
Source: PLoS One. 2017 Dec 14;12(12):e0189483. doi: 10.1371/journal.pone.0189483 (PMC5730181; doi:10.1371/journal.pone.0189483)
Supplement: S3 Table — The effect of the depicted mutations in the ability to grow in liquid minimal medium (CSK) supplemented with a single carbon/energy source is represented by ‘++’ (normal growth rate), ‘+’ (slightly to moderately decreased growth rate), and ‘-’ (strongly decreased growth rate or no growth). ‘NA’, no data available. * Grown in the presence of 1mM IPTG. Wild-type is B. subtilis 168T+, and all other strains have a 168T+ background. (DOCX) [file pone.0189483.s003.docx]

**Table S3 – Growth of B. subtilis strains in the presence of different carbon sources.** The effect of the depicted mutations in the ability to grow in liquid minimal medium (CSK) supplemented with a single carbon/energy source is represented by ‘++’ (normal growth rate), ‘+’ (slightly to moderately decreased growth rate), and ‘-’ (strongly decreased growth rate or no growth). ‘NA’, no data available. * Grown in the presence of 1mM IPTG. Wild-type is B. subtilis 168T^+^, and all other strains have a 168T^+^ background.

|  | **Wild-type** | **Δ*msmX*** | **Δ*yurJ*** | *** Δ*msmX* Phyper-spank-*yurJ*** | **Δ*araNPQ*** | **Δ*cycB*** | **Δ*cycB* Δ*araNPQ*** | **Δ*cycB* *araA**E305A** | **Δ*galK*** | **Δ*galK* Δ*araNPQ*** | **Δ*yesOPQ*** | **Δ*ytcQ*** | **Δ*yesOPQ* Δ*ytcQ*** |
| --- | --- | --- | --- | --- | --- | --- | --- | --- | --- | --- | --- | --- | --- |
| **Glucose** | ++ | ++ | ++ | ++ | ++ | ++ | ++ | ++ | ++ | ++ | ++ | ++ | ++ |
| **Arabinose** | ++ | ++ | ++ | ++ | ++ | NA | NA | - | NA | NA | NA | NA | NA |
| **Galacturonic Acid** | ++ | ++ | NA | NA | NA | NA | NA | NA | NA | NA | ++ | NA | NA |
| **Arabinotriose** | ++ | - | ++ | ++ | - | NA | NA | NA | NA | NA | NA | NA | NA |
| **Pectin** | ++ | - | ++ | NA | ++ | NA | NA | NA | NA | NA | ++ | ++ | ++ |
| **Arabinan** | ++ | - | ++ | NA | + | + | - | NA | + | - | NA | NA | NA |
| **Galactan** | ++ | - | NA | NA | + | + | - | - | + | - | NA | NA | NA |
| **Rhamnogalacturonan** | ++ | + | NA | NA | NA | NA | NA | NA | NA | NA | + | + | + |
| **Polygalacturonan** | ++ | + | NA | NA | NA | NA | NA | NA | NA | NA | + | + | + |
