# Supplementary figures and images for: The MsmX ATPase plays a crucial role in pectin mobilization by Bacillus subtilis
Source: PLoS One. 2017 Dec 14;12(12):e0189483. doi: 10.1371/journal.pone.0189483 (PMC5730181; doi:10.1371/journal.pone.0189483)

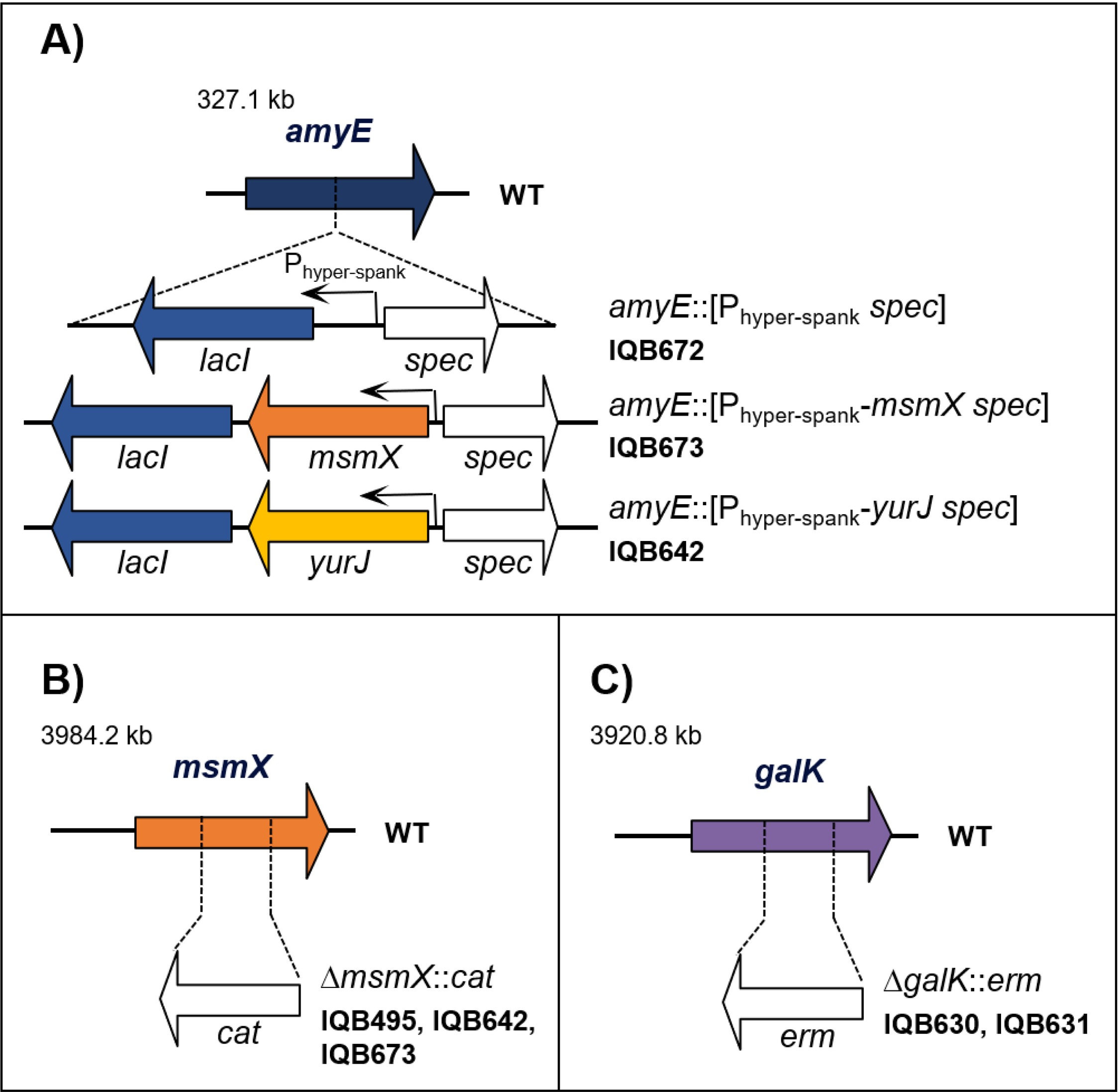

Supplement: S1 Fig — The location of the three regions is indicated in kilobase pairs. The genes are represented by arrows pointing in the direction of transcription. The constructs bearing the mutations used in this work are displayed below each region, and the strains harboring each mutation are indicated in front of the construct. The mutations generated by the ectopic insertion of msmX or yurJ under control of an inducible promoter are represented at the amyE locus. The insertion-deletion mutation created by a deletion in msmX followed by the insertion of a chloramphenicol resistance cassette (cat) is shown below the msmX locus. The insertion-deletion mutation created by a deletion in galK followed by the insertion of an erythromycin resistance cassette (erm) is shown below the galK locus. All constructions are described in Materials and Methods and supporting information. (TIF) [file pone.0189483.s004.tif]

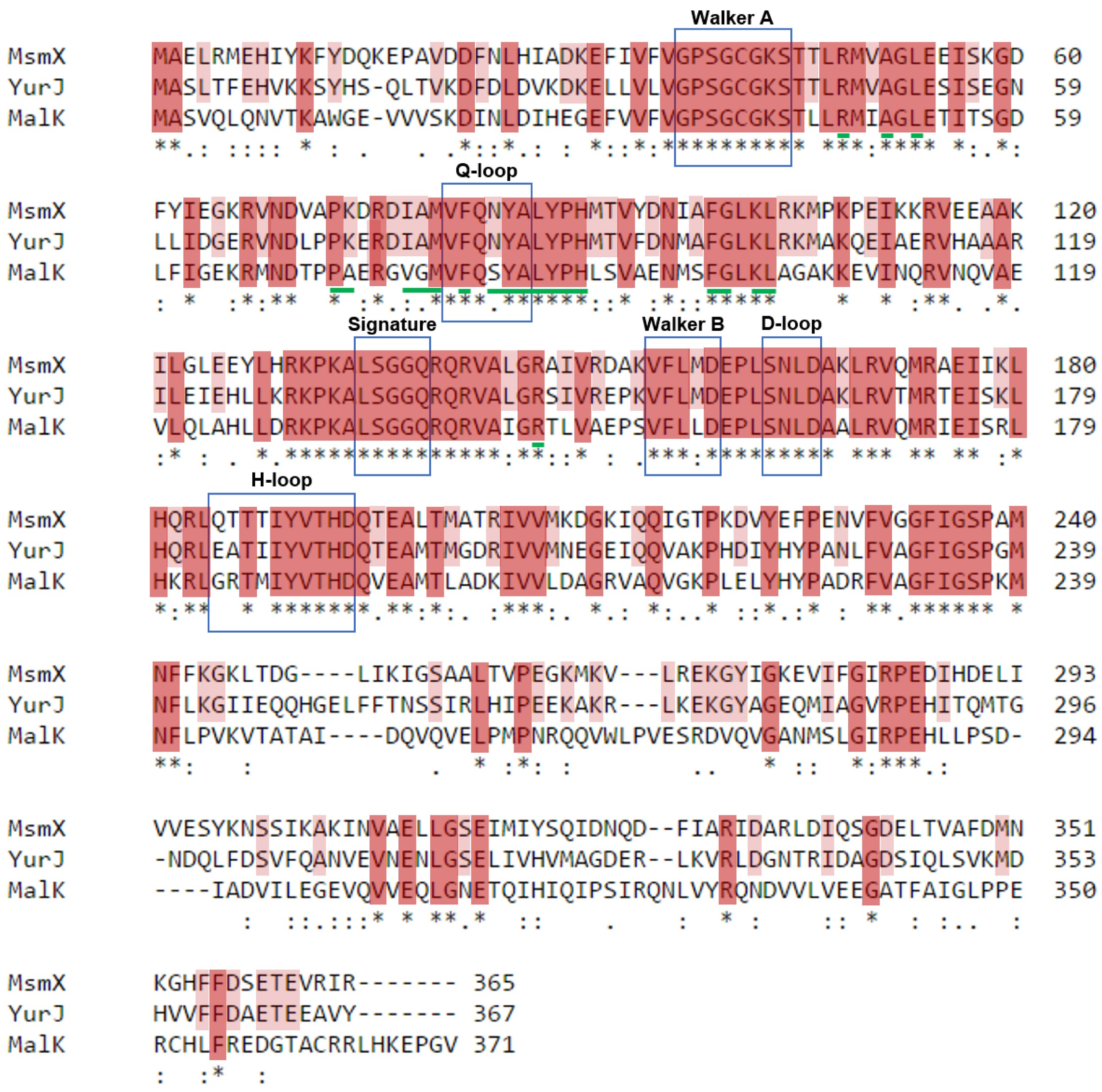

Supplement: S2 Fig — The alignment between B. subtilis MsmX and YurJ, and E. coli MalK was obtained using Clustal Omega (http://www.ebi.ac.uk/Tools/msa/clustalo/). Identical (´*´) and similar (´.´ or ´:´) amino acids are indicated. Gaps in the amino acid sequences inserted for alignment optimization are indicated by a dash (–). Conserved ABC ATPase motifs (Walker A, Q loop, Signature motif, Walker B, D loop, and H loop) are boxed. Identical residues between the three ATPases are highlighted in red. Identical residues between MsmX and YurJ are highlighted in pink. MalK residues involved in interactions with the TMDs of the E. coli maltose transporter are underlined in green. Accession numbers: MsmX (P94360), YurJ (O32151), and MalK (P68187). (TIF) [file pone.0189483.s005.tif]

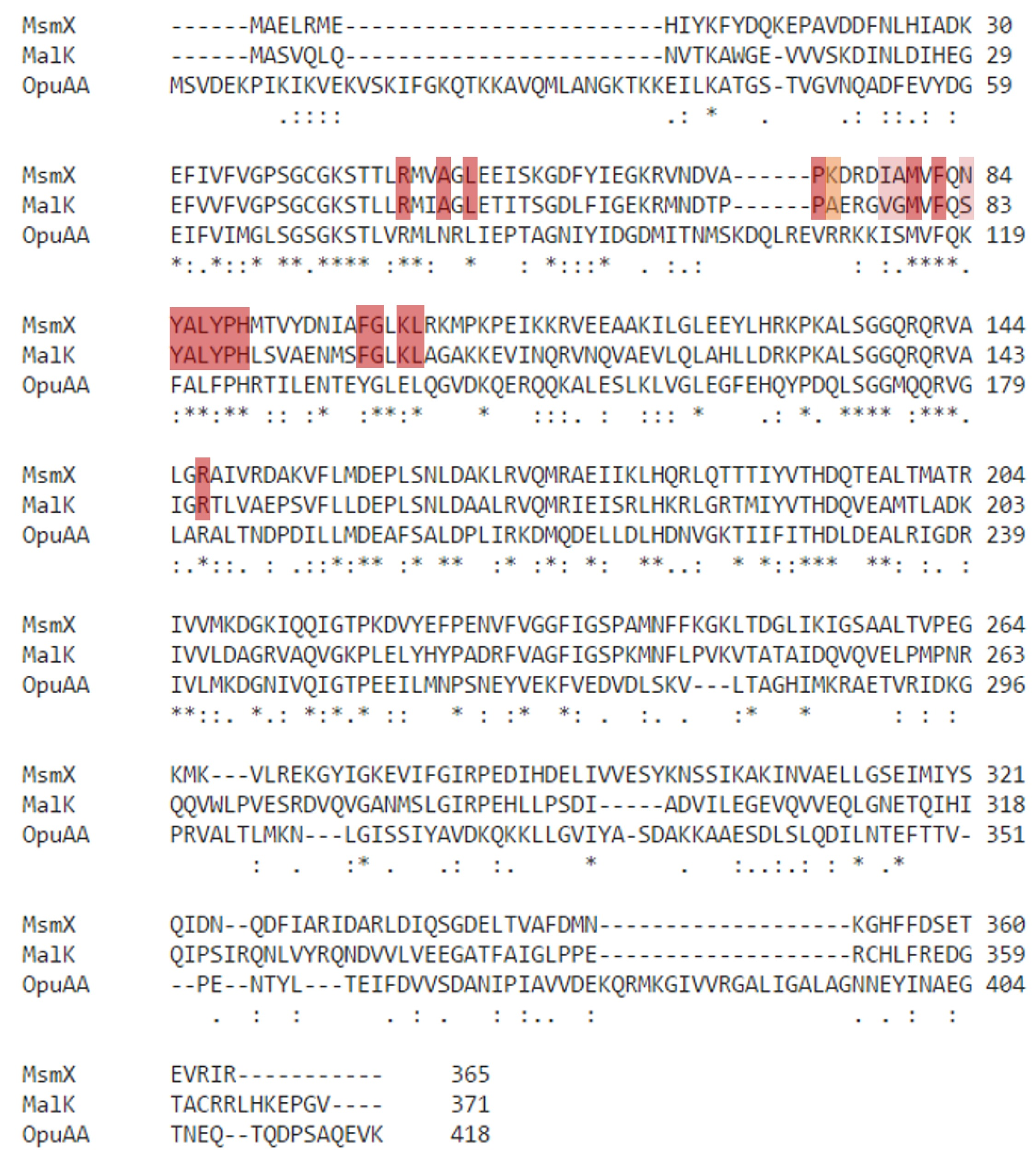

Supplement: S3 Fig — The alignment between B. subtilis MsmX and OpuAA, and E. coli MalK was obtained using Clustal Omega (http://www.ebi.ac.uk/Tools/msa/clustalo/). Identical (´*´) and similar (´.´ or ´:´) amino acids are indicated. Gaps in the amino acid sequences inserted for alignment optimization are indicated by a dash (–). MalK residues involved in interactions with the TMDs of the E. coli maltose transporter are highlighted in red (identical in MsmX), pink (similar in MsmX), or orange (not conserved in MsmX). Accession numbers: MsmX (P94360), MalK (P68187), and OpuAA (P46920). (TIF) [file pone.0189483.s006.tif]
