# Supplementary material for: The MsmX ATPase plays a crucial role in pectin mobilization by Bacillus subtilis
Source: PLoS One. 2017 Dec 14;12(12):e0189483. doi: 10.1371/journal.pone.0189483 (PMC5730181; doi:10.1371/journal.pone.0189483)
Supplement: S1 Appendix — (DOCX) [file pone.0189483.s007.docx]

**Construction of plasmids and *B. subtilis* strains**

All plasmids and primers described in this section are listed in Table S1 and Table S2, respectively. For the construction of a *B. subtilis* *yurJ* deletion mutant, the regions upstream and downstream of the *yurJ* gene were independently amplified by PCR, from chromosomal DNA of *B. subtilis* 168T^+^, with primers ARA517 and ARA518 (PCR1) and ARA519 and ARA520 (PCR2), respectively. The products from PCR1 and PCR2 were joined by overlapping PCR using primers ARA517 and ARA520 (Table S2). The final product was digested with EcoRV and the resulting 1620 bp fragment cloned into pMAD digested with SmaI and subsequently dephosphorylated. The obtained plasmid, pMJ14, was used for the generation of a clean deletion of *yurJ* in the chromosome of *B. subtilis* strain 168T^+^, following the procedure described by Arnaud *et al*. [21]. The deletion was confirmed by sequencing and the new strain named IQB618. To construct a *B. subtilis* strain with a copy of *msmX* under the control of an IPTG-inducible promoter, in the *amyE* locus of the *B. subtilis* chromosome, this gene was amplified by PCR from *B. subtilis* 168T^+^ chromosomal DNA using primers ARA741 and ARA742 (Table S2). The amplification product was digested with SalI and SphI and the resulting 1224 bp DNA fragment was cloned into pDR111 (Table S1) digested with the same restriction enzymes. The obtained plasmid, pAM4, and pDR111 were linearized with ScaI and used for the transformation of *B. subtilis* IQB495, yielding strains IQB673 and IQB672, respectively. To construct a *B. subtilis* strain with a copy of *yurJ* under the control of an IPTG-inducible promoter, in the *amyE* locus of the *B. subtilis* chromosome, this gene was amplified by PCR from *B. subtilis* 168T^+^ chromosomal DNA using primers ARA820 and ARA821 (Table S2). The amplification product was digested with SalI and SphI and the resulting 1222 bp DNA fragment was cloned into pDR111 (Table S1) digested with the same restriction enzymes. The obtained plasmid, pMJ40, was linearized with ScaI and used for the transformation of *B. subtilis* IQB495, yielding strain IQB642. For the construction of a *B. subtilis* strain with a C-terminal His_6_-tag fused to YurJ, the 3’ end of *yurJ* was amplified by PCR, from chromosomal DNA of *B. subtilis* 168T^+^, using primers ARA831 and ARA832 (Table S2). The obtained product of 819 bp was phosphorylated and subcloned into pMS38 (Table S1) digested with EcoRV and subsequently dephosphorylated, resulting in pMJ43. This plasmid was used for the transformation of *B. subtilis* 168T^+^. The fusion of the His_6_-tag to *yurJ* was confirmed by sequencing and the new strain was named IQB644. To construct a *B. subtilis* strain with a C-terminal His_6_-tag fused to YurJ, in a MsmX-null background, plasmid pCm::Sp was used to switch the chloramphenicol resistance marker in strain IQB495 (Δ*msmX*::*cat*) by a spectinomycin resistance marker. The resulting strain, IQB650, was then transformed with pMJ43 to create the *yurJ-his_6_-tag* allele (IQB651; Δ*msmX*::*spec* *yurJ*-His_6_ *cat*). For the generation of *B. subtilis* mutant strains with in-frame deletions of *yesOPQ* and *ytcQ*, plasmids pMJ18 and pMJ33 were constructed. The regions upstream of the *yesO* gene and downstream of the *yesQ* gene were independently amplified by PCR, from chromosomal DNA of *B. subtilis* 168T^+^, with primers ARA642 and ARA652 (PCR3) and ARA653 and ARA645 (PCR4), respectively. The products from PCR3 and PCR4 were joined by overlapping PCR using primers ARA642 and ARA645 (Table S2). The final product was digested with MluI and the resulting 1586 bp DNA fragment was cloned into pMAD digested with SmaI and MluI. The obtained plasmid, pMJ18, was used for the generation of clean deletions of *yesOPQ* in the chromosome of *B. subtilis* strains 168T^+^ and IQB611 (Table 1), following the published procedure described by Arnaud *et al*., 2004 [21]. These deletions were confirmed by sequencing and the new strains were named IQB628 and IQB629, respectively. The regions upstream and downstream of the *ytcQ* gene were independently amplified by PCR, from chromosomal DNA of *B. subtilis* 168T^+^, with primers ARA733 and ARA734 (PCR5) and ARA735 and ARA736 (PCR6), respectively. The products from PCR5 and PCR6 were joined by overlapping PCR using primers ARA733 and ARA736 (Table S2). The final product was digested with MluI and the resulting 1541 bp DNA fragment was cloned into pMAD digested with SmaI and MluI. The obtained plasmid, pMJ33, was used for the generation of clean deletions of *ytcQ* in the chromosome of *B. subtilis* strains 168T^+^ and IQB628, following the published procedure described by Arnaud *et al*., 2004 [21]. These deletions were confirmed by sequencing and the new strains were named IQB632 and IQB633, respectively. For the generation of *B. subtilis* mutant strains with an in-frame deletion of *cycB*, plasmid pMJ38 was constructed. The regions upstream and downstream of the *cycB* gene were independently amplified by PCR, from chromosomal DNA of *B. subtilis* 168T^+^, with primers ARA714 and ARA779 (PCR7) and ARA780 and ARA781 (PCR8), respectively. The products from PCR7 and PCR8 were joined by overlapping PCR using primers ARA714 and ARA781 (Table S2). The final product was digested with MluI and the resulting 1579 bp DNA fragment was cloned into pMAD digested with SmaI and MluI. The obtained plasmid, pMJ38, was used for the generation of clean deletions of *cycB* in the chromosome of *B. subtilis* strains 168T^+^ and IQB611, following the published procedure described by Arnaud *et al*., 2004 [20]. These deletions were confirmed by sequencing and the new strains were named IQB638 and IQB639, respectively (Table 1). To construct a *B. subtilis galK* insertion-deletion mutant, *galK* was amplified by PCR from chromosomal DNA of *B. subtilis* 168T^+^ using primers ARA718 and ARA719 (Table S2), and the obtained product was digested with SacI and KpnI. The resulting 1300 bp DNA fragment was cloned into the phagemid pBluescript II KS(+) (Table S1), also digested with SacI and KpnI, yielding pMJ31. An erythromycin resistance (Erm^R^) cassette was obtained by the digestion of pMJ23 with NaeI and EcoRV and subcloned between the StuI sites of pMJ31, resulting in pMJ32. pMJ23 is a pBluescript II KS(+) derivative carrying an Erm^R^ cassette obtained from the digestion of pJL1 with BamHI and PstI. pJL1 is a pLitmus29 derivative carrying an Erm^R^ cassette, obtained from the digestion of pDG1731 with SmaI and NaeI, cloned in its EcoRV site. pMJ32 was linearized with ScaI and used to transform *B. subtilis* 168T^+^ and IQB611 yielding strains IQB630 and IQB631, respectively (Table 1). For the construction of a *B. subtilis* strain with a single mutation leading to a E305A amino acid substitution in the AraA protein, *araA* was amplified by PCR, from *B. subtilis* 168T^+^ chromosomal DNA, with primers ARA55 and ARA573 (Table S2). The obtained product was digested with EcoRI and KpnI and the resulting 2313 bp fragment cloned into pUC18 digested with the same restriction enzymes. The obtained pUC18::*araA* was used as template for a PCR with primers ARA566 and ARA567 (Table S2). The resulting pUC18::*araA*(E305A) was digested with SalI and EcoRI and the 2328 bp fragment with the mutagenized *araA* gene was subcloned into pMAD digested with the same restriction enzymes, yielding pLG31. This new plasmid was used for the generation of a mutation leading to an E305A amino acid substitution in AraA. *B. subtilis* strain IQB638 was transformed following the published procedure described by Arnaud *et al*., 2004 [21]. The mutation was confirmed by sequencing and the new strain was named IQB641. For the generation of single E208A, E205A and D213A substitutions in AraP of *B. subtilis*, pLB1, pMJ25 and pMJ26 were constructed. For the E208A substitution two partially overlapping regions upstream and downstream of the desired codon were independently amplified by PCR, from *B. subtilis* 168T^+^ chromosomal DNA, using primers ARA630 and ARA631 (PCR9) and primers ARA632 and ARA633 (PCR10), respectively. The products from PCR9 and PCR10 were joined by overlapping PCR using primers ARA630 and ARA633 (Table S2). The obtained product was digested with BamHI and NcoI and the resulting 1595 bp DNA fragment was cloned into pMAD digested with the same restriction enzymes, yielding pLB1. A similar approach was used for the E205A and D213A substitutions. For the E205A substitution the initial upstream and downstream amplifications were performed with primers ARA630 and ARA667 (PCR11) and primers ARA668 and ARA633 (PCR12), respectively. The products from PCR11 and PCR12 were joined by overlapping PCR using primers ARA630 and ARA633 (Table S2). The obtained product was digested with BamHI and NcoI and the resulting 1595 bp DNA fragment was cloned into pMAD digested with the same restriction enzymes, yielding pMJ25. For the D213A substitution the initial upstream and downstream amplifications were performed with primers ARA630 and ARA669 (PCR13) and primers ARA670 and ARA633 (PCR14), respectively. The products from PCR13 and PCR14 were joined by overlapping PCR using primers ARA630 and ARA633 (Table S2). The obtained product was digested with BamHI and NcoI and the resulting 1595 bp DNA fragment was cloned into pMAD digested with the same restriction enzymes, yielding pMJ26. The obtained plasmids, pLB1, pMJ25 and pMJ26, were used for the generation of mutations in *araP* in the chromosome of *B. subtilis* 168T^+^, leading to single amino acid substitutions in the AraP protein, following the published procedure described by Arnaud *et al*., 2004 [21]. The E208A, E205A and D213A substitutions were confirmed by sequencing and the new strains were named IQB623, IQB624 and IQB625, respectively. For the generation of a single D180A substitution in AraQ of *B. subtilis*, pMJ27 was constructed. Two independent and partially overlapping regions upstream and downstream of the desired codon were amplified by PCR, from *B. subtilis* 168T^+^ chromosomal DNA, using primers ARA634 and ARA671 (PCR15) and primers ARA672 and ARA637 (PCR16), respectively. The products from PCR15 and PCR16 were joined by overlapping PCR using primers ARA634 and ARA637 (Table S2). The obtained product was digested with BamHI and NcoI and the resulting 1489 bp DNA fragment was cloned into pMAD digested with the same restriction enzymes, resulting in pMJ27. The obtained plasmid was used for the generation of a mutation in *araQ* in the chromosome of *B. subtilis* 168T^+^, leading to a single D180A amino acid substitution in the AraQ protein, following the published procedure described by Arnaud *et al*., 2004 [21]. The amino acid substitution was confirmed by sequencing and the new strain was named IQB626. For the construction of a *B. subtilis* strain with an in-frame deletion of the 4 codons at the 3’ end of *araQ*, the regions upstream and downstream of the target codons were independently amplified by PCR, from *B. subtilis* 168T^+^ chromosomal DNA, using primers ARA673 and ARA674 (PCR17) and primers ARA675 and ARA676 (PCR18), respectively. The products from PCR17 and PCR18 were joined by overlapping PCR using primers ARA673 and ARA676 (Table S2). The obtained product was digested with MluI and the resulting 1605 bp DNA fragment was cloned into pMAD digested with SmaI and MluI, yielding pMJ28. The obtained plasmid was used for the generation of a clean deletion of 4 codons of *araQ* in the chromosome of *B. subtilis* strains 168T^+^, following the published procedure described by Arnaud *et al*., 2004 [21]. This deletion was confirmed by sequencing and the new strain was named IQB627. For the fusion of a C-terminal Histidine-tag to MsmX, a DNA fragment containing part of the *msmX* gene was obtained, from chromosomal DNA of *B. subtilis* 168T^+^, by PCR with primers ARA544 and ARA545 (Table S2) and digestion with NdeI and XhoI. The resulting 829 bp was cloned into pET30a(+) (Table S1) digested with the same enzymes, yielding pMJ21. A 270 bp DNA fragment containing the 5’ end of the *msmX* gene (amplified with primers ARA544 and ARA545 and digested with NdeI) was subcloned into NdeI-digested pMJ21, resulting in pMJ22. To construct a *B. subtilis* strain with a C-terminal His_6_-tag fused to MsmX, pMJ22 was digested with SspI and the resulting 1329 bp DNA fragment, containing the 3’ end of *msmX* fused to the histidine tag, was subcloned into pMS38 (Table S1), resulting in pGS1. This plasmid was used for the transformation of *B. subtilis* 168T^+^. The fusion of the His_6_-tag to *msmX* was confirmed by sequencing and the new strain was named IQB622. The transformations of *B. subtilis* strains were performed according to the method described by Anagnostopoulos and Spizizen [22].
